# PCA-PI-PLS-ABS: A new approach for uncovering directional heterogeneity in PLS-SEM

**DOI:** 10.1016/j.mex.2026.104177

**Published:** 2026-09-25

**Authors:** Angga Dwi Mulyanto, Bambang Widjanarko Otok, Jerry Dwi Trijoyo Purnomo

**Affiliations:** a Department of Statistics, Faculty of Science and Data Analytics, Institut Teknologi Sepuluh Nopember, Surabaya, 60111, Indonesia; b Department of Mathematics, Faculty of Science and Technology, Universitas Islam Negeri Maulana Malik Ibrahim Malang, Malang, 65144, Indonesia

**Keywords:** Simpson’s paradox, Power iteration, Hierarchical agglomerative clustering, Geometric transformation, Latent variable path modeling, Segmentation

## Abstract

Standard Partial Least Squares Structural Equation Modeling (PLS-SEM) methods often fail to detect significant structural relationships when Simpson's Paradox is present in the data (e.g., when one subgroup has a positive effect while another subgroup has a negative effect). In such cases the subgroup effects may be cancelled in the aggregate (global) model, so that the pooled coefficient describes neither subgroup. This article introduces PCA-PI-PLS-ABS, a new approach designed to efficiently reveal such directional heterogeneity. Monte Carlo simulation validation shows that this method successfully separates data groups with opposite directions (β_A_ = 1.0 and β_B_ = -1.0) with a segmentation accuracy of 94%, where the standard global model fails to capture this relationship.

This method offers a solution with the following key characteristics:

Integrating Power Iteration-based Principal Component Analysis (PCA) for faster computational estimation of outer weights.

Applies Angle-Based Segmentation (ABS) using geometric transformations to group observations based on the similarity of their structural relationships.

Capable of separating sub-populations with mutually opposing path coefficients.

The method is deliberately targeted: it separates subgroups whose structural relationships differ in direction and is not intended to separate subgroups that share a direction and differ only in effect size. We characterise this boundary explicitly through a calibrated minimum detectable angular separation, so that researchers can judge in advance whether the procedure is feasible for their own data.

## Graphical abstract

**Figure:**
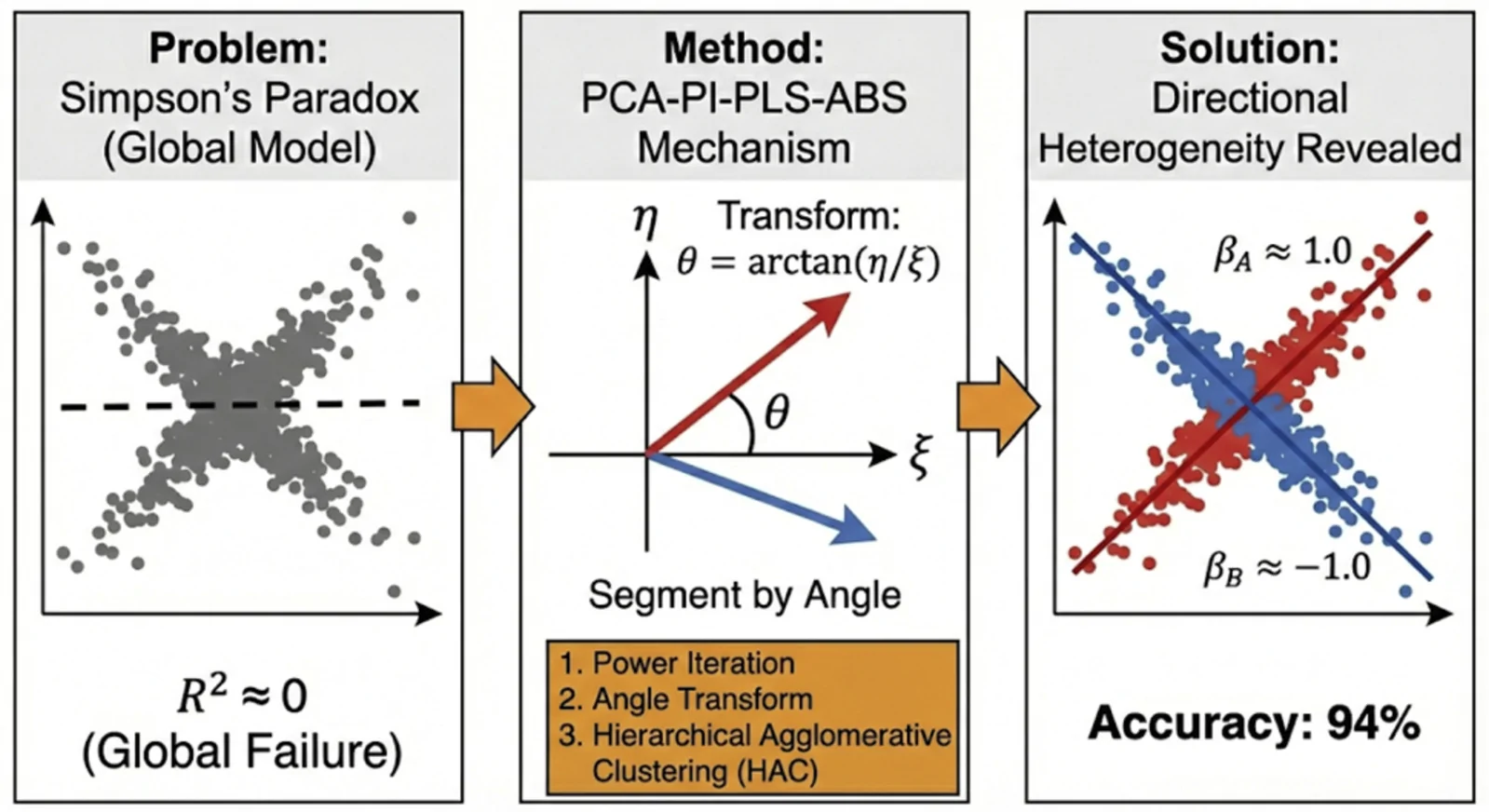

**Specifications table**

**Table:** 

**Subject area**	Mathematics and Statistics
**More specific subject area**	Statistics; Multivariate Analysis; Structural Equation Modeling; Segmentation; Computational Statistics
**Name of your method**	PCA-PI-PLS-ABS
**Name and reference of original method**	PLS Algorithm as described in: 1. Lohmöller, J. (1989) Latent Variable Path Modeling with Partial Least Squares. Berlin: Springer. 2. Hair Jr, J.F., Hult, G.T.M., Ringle, C.M., Sarstedt, M., Danks, N.P., & Ray, S. (2021) Partial Least Squares Structural Equation Modeling (PLS-SEM) using R. Cham: Springer. Power Iteration as described in: 3. Sauer, T. (2019) Numerical Analysis. Third Edition. Hoboken: Pearson. Hierarchical Agglomerative Clustering as described in: 4. Miyamoto, S. (2022) Behaviorametrics: Quantitative Approaches to Human Behavior Volume 15 Theory of Agglomerative Hierarchical Clustering. Singapore: Springer
**Resource availability**	The R source code provided corresponds to the first simulation scenario (Simulation 1), which serves as the base template. Parameters within this script can be adjusted to reproduce the subsequent scenarios (Simulations 2–17). The raw output logs for all scenarios are provided in the supplementary ZIP archive. Additionally, the computational report is available at https://rpubs.com/anggadwimulyanto/PCA-PI-PLS-ABS.

## Background

PLS-SEM has been well applied in different other areas like social science [1], economics [2], and health [3] to model a complex relationship among latent variables. Standard PLS-SEM however makes the assumption that the population is homogeneous i.e. a single model of the world can be applied to all the observations. As a matter of fact, empirical data are regularly, unobserved heterogeneous, with various sub-populations having dissimilar structural relationships (e.g., the effects of predictor variables on outcomes might differ in various regions).

Existing methods for handling this heterogeneity, such as FIMIX-PLS or REBUS-PLS, can be computationally intensive or sensitive to initialization [[4], [5], [6]]. Furthermore, error or distance-based segmentation often fails when data points cluster around the mean (the "zero crossing" problem). This makes it difficult to distinguish between positive and negative path relationships (Simpson's Paradox phenomenon) [7].

In order to overcome these difficulties, we suggest PCA-PI-PLS-ABS method. This method presents two key variations (1) the fast and stable estimation of outer weights with Power Iteration and (2) the Angle-based transformation of the segmentation. This geometrical transformation is necessary to ensure that heterogeneity is identified based on the direction (slope) of relationship and not on the score magnitude, which is a powerful tool in identifying the groups of opposite structural pathway.

## Method details

### Partial least squares structural equation modeling (PLS-SEM)

PLS-SEM is a non-parametric multivariate statistical technique [8,9] that is utilized to test structural connections between the manifest variables (indicators) and latent variables (unobserved constructs) [[10], [11], [12]]. PLS-SEM in contrast to Covariance-Based SEM, seeks to maximize the amount of endogenous latent variables that are explained by the sample ($R^{2}$). This algorithm operates by approximating latent variables on the basis of their indicators by a linear combination. PLS-SEM has measurement models and structural models [8].

The measurement model is a definition of the correlation between latent variables and indicators of latent variables. PLS framework has two relationship modes reflective (Mode A) and formative (Mode B) [8]. Using mathematics, the reflective model which assumes indicators to be dependent on latent variables is expressed as [6,13]:

(1) $$\begin{array}{c} x_{jh}=\lambda_{jh}\xi_{j}+\varepsilon_{jh} \end{array}$$

where:

• $x_{jh}$ is the h-th indicator of the exogenous latent variable $\xi_{j}$.
• $\lambda_{jh}$ is *the outer loading* that indicates the correlation between the indicator and its latent variable.
• $\varepsilon_{jh}$ is *the measurement error* or measurement residual.

Meanwhile, for the endogenous latent variable η, the equation is:

(2) $$\begin{array}{c} y_{kg}=\lambda_{kg}\eta_{k}+\varepsilon_{kg} \end{array}$$

Where $y_{kg}$ is the g-th indicator for the endogenous latent variable $\eta_{k}$.

In parameter estimation, PLS calculates the latent variable score as the weighted sum of its indicators ( $x_{jh}$ see Eq. (1) dan $y_{kg}$ see Eq. (2)), expressed as [14]:

(3) $$\xi_{j}=\sum_{h=1}^{H}w_{jh}x_{jh}$$

(4) $$\begin{array}{c} \eta_{k}=\sum_{g=1}^{G}w_{kg}y_{kg} \end{array}$$

Where w is the outer weight calculated by a given method, e.g. centroid, factor, or path.

The structural model explains the cause and effect relationship of latent variables. This model defines the prediction of the endogenous latent variables (η) by exogenous latent variables (ξ). Mathematically, this structural relationship can be described in a form of a set of linear equations as in Eq. (5) [14].

(5) $$\begin{array}{c} \eta_{k}=\sum_{j=1}^{J}\beta_{kj}\xi_{j}+\sum_{l=1}^{L}\gamma_{kl}\eta_{l}+\zeta_{k} \end{array}$$

where:

• $\beta_{kj}$ is the path coefficient that connects the exogenous variable $\xi_{j}$ (see Eq. (3)) to the endogenous variable $\eta_{k}$ (see Eq. (4)).
• $\gamma_{kl}$ is the path coefficient connecting other endogenous variables $\eta_{l}$ to $\eta_{k}$.
• $\zeta_{k}$ is the structural residual (inner residual), which is assumed to have a mean of zero and to be uncorrelated with the predictor variables.

The path coefficients (β and γ) in Eq. (5) are estimated using OLS (*Ordinary Least Squares*) regression after the latent variable scores (ξ and η) are obtained from the PLS algorithm iteration stage.

### PLS algorithm

Prior to the PCA-PI-PLS-ABS algorithm's introduction, it would be better to first introduce the underlying basic algorithm, namely the PLS algorithm. This algorithm operates by estimating latent variable scores as weighted linear combinations of their indicators. The comprehensive stepwise mechanism, which serves as the baseline for this study [15,16], is presented in Table 1.

**Table 1:** PLS algorithm.

1. Data Preparation and Initialization Stage 1.1. Designing an output in the form of a list that is still empty. 1.2. Calculate the number of indicators 1.3. Calculate the number of latent variables 1.4. Calculate the number of observations 1.5. Standardize the data in matrix form. 1.6. Mapping indicators to latent variables 1.7. Mapping the outer model type 1.8. Converting standardization into list form 1.9. Initializing weights with random values 1.10. Calculating f values 1.11. Calculating outer weight 1.12. Outside Approximation
2. Interaction stages for estimating outer weight and latent variable scores 2.1. Setting the result check to false 2.2. Setting iteration to iteration 1 2.3. While loop (stop when result check is true) 2.3.1. Calculating Inner Weight (there are 3 approaches: Centroid, Factor, or Path) 2.3.2. Inside Approximation 2.3.3. Outer Weight 2.3.3.1. Calculating the new weight value 2.3.3.2. Checking: if the difference between all new weights and the old weights is less than the predefined threshold ($1\;\times 10^{-5}$ ), the loop terminates; otherwise, the loop continues 2.3.3.3. Add the iteration value (+1) and replace the old weight value with the new weight 2.3.3.4. Calculate the f value 2.3.3.5. Calculating the outer weight 2.3.4. Outside Approximation 2.4. Setting the Final Outer Weight and Final Latent Variable Score
3. Path Coefficient and Outer Loading Estimation Steps 3.1. Creating a list of exogenous columns 3.2. Creating the exogenous matrix 3.3. Calculating Path Coefficients 3.4. Calculating Outer Loadings 3.5. Calculating R^2^
4. Location parameter estimation stages 4.1. Calculating m 4.2. Calculating nx 4.3. Calculating p_0_ 4.4. Calculating b_0_

### PCA-PI-PLS algorithm

The PCA-PI-PLS Algorithm presented in Table 2 is a modification of the PLS algorithm by replacing procedure 1.9 in Table 1 with PCA weighting using the Power Iteration approach. Consequently, Step 2 in Table 1 is not necessary. Unlike the conventional PCA approach, which uses the SVD method [[17], [18], [19]] that outputs all eigen values (PC_1_ , PC_2_ , …, PC_k_), the Power Iteration method focuses only on the dominant eigen value, namely PC_1_ thereby reducing the calculation time [20,21].

**Table 2:** PCA-PI-PLS algorithm.

1. Data Preparation and Initialization Steps 1.1. Design an output in the form of a list, but still empty. 1.2. Calculate the number of indicators 1.3. Calculate the number of latent variables 1.4. Calculate the number of observations 1.5. Standardize the data in matrix form. 1.6. Mapping indicators to latent variables 1.7. Mapping the outer model type 1.8. Converting standardization into list form **1.9. Weight of PCA-PI** 1.10. Calculating the f value 1.11. Calculating outer weights 1.12. Outside approximation
2. Steps for Estimating Path Coefficients and Outer Loadings 2.1. Creating a list of exogenous columns 2.2. Creating the exogenous matrix 2.3. Calculating Path Coefficients 2.4. Calculating Outer Loadings 2.5. Calculating R^2^
3. Location parameter estimation stages 3.1. Calculating m 3.2. Calculating nx 3.3. Calculating p_0_ 3.4. Calculating b_0_

In PCA-PI-PLS step 1.9, to obtain the weight ($w_{k}$ ), the eigenvector ($\nu_{k}$ ) resulting from the Power Iteration method as in Eq. (5.1)is used.

(5.1) $$\begin{array}{c} w_{k}=\left[\begin{array}{c} w_{k1}\\ w_{k2}\\ \vdots \\ w_{kj_{k}}\\ \vdots \\ w_{kJ_{k}} \end{array}\right]=\nu_{k} \end{array}$$

The first step in the Power Iteration method, as in Eq. (6), is to initialize the vector $g_{0k}$ with all values of 1, where k=1,2,…,K and K are the number of latent variables.

(6) $$\begin{array}{c} g_{0k}=\left[\begin{array}{c} g_{10k}\\ g_{20k}\\ \vdots \\ g_{j_{k}0k}\\ \vdots \\ g_{J_{k}0k} \end{array}\right]=\left[\begin{array}{c} 1\\ 1\\ \vdots \\ 1\\ \vdots \\ 1 \end{array}\right] \end{array}$$

Then, continue with the following iteration process until convergence [22]:

1) Vector Normalization
  $$\nu_{\left(q-1\right)k}=\frac{g_{\left(q-1\right)k}}{∥g_{\left(q-1\right)k}{∥}_{2}}$$
2) Matrix-Vector Multiplication
  $$a_{\mathrm{qk}}={\hat{\Sigma }}_{k}\nu_{\left(q-1\right)k}$$
3) Calculate Eigenvalues
  $$\psi_{\text{qk}}=\nu_{\left(q-1\right)k}^{{}^{\prime }}{\hat{\Sigma }}_{k}\nu_{\left(q-1\right)k}$$
where ${\hat{\Sigma }}_{k}$ is the covariance matrix of the standardized data.
4) Calculate Eigenvector
  $$\nu_{\text{qk}}=\frac{a_{\mathrm{qk}}}{∥a_{\mathrm{qk}}{∥}_{2}}$$

Repeat steps 1 through 4. The iteration stops when it converges by satisfying the following criteria:

$$∥\nu_{\text{qk}}-\nu_{\left(q-1\right)k}{∥}_{2}<0.000001$$

The eigen value and eigen vector at the time of convergence are the final values for the eigen value and eigen vector.

$$\nu_{k}=\nu_{\text{qk}}$$

$$\psi_{k}=\psi_{\text{qk}}$$

This approach provides weight values fairly quickly compared to other methods for finding eigenvectors, because Power Iteration focuses only on dominant eigenvectors.

### PCA-PI-PLS-ABS algorithm

The PCA-PI-PLS algorithm presented in Table 2 has a limitation to global estimation of the model, and that of the population is homogeneous. Population homogeneity refers to the uniformity within a population. When a population is homogeneous, the likelihood of Simpson’s Paradox occurring is reduced because the aggregated data is more likely to reflect the true trends within the subgroups. Conversely, in heterogeneous populations, where subgroups exhibit different behaviors or characteristics, Simpson’s Paradox is more likely to occur [[23], [24], [25]]. The extended PCA-PI-PLS-ABS algorithm is provided to help deal with the issue of unobserved heterogeneity (as is the case with Simpson’s Paradox [7]). This hybrid algorithm combines the effective Power Iteration with an Angle-Based Segmentation (ABS) thus it is possible to automatically define and isolate sub-populations, which are structurally opposite to each other.

Following step 4, structural heterogeneity (angle-based) can be detected by calculating the θ value for each observation i as in Eq. (7).

(7) $$\begin{array}{c} \theta_{i}=\arctan\left(\frac{{\hat{\eta }}_{i}}{{\hat{\xi }}_{i}+\epsilon }\right) \end{array}$$

Where i=1,2,…,n, and n are the number of observations/samples. Meanwhile, ${\hat{\eta }}_{i}$ is the latent score of the endogenous variable for the i-th observation, and ${\hat{\xi }}_{i}$ is the latent score of the exogenous variable for the i-th observation. Meanwhile, $\epsilon =10^{-6}$ is a small number added to prevent division by zero. Observations that follow a positive path relationship will have a positive θ value (e.g.,$\approx 45^{o}$ ), while a negative relationship will result in a negative θ.

The transformation in Eq. (7) uses the ordinary arctangent rather than the two-argument atan2 function, and the choice is deliberate. What the segmentation groups is the orientation of the structural relationship, that is the slope β of the line η=βξ, and an orientation is an axis rather than a ray: the line occupies two opposite quadrants simultaneously, so an observation $\left({\overset{ˆ}{\xi }}_{i},{\overset{ˆ}{\eta }}_{i}\right)$ and its antipode $\left(-{\overset{ˆ}{\xi }}_{i},-{\overset{ˆ}{\eta }}_{i}\right)$ lie on the same line, represent the same β, and must receive the same angle. This is the defining property of axial data [26,27], of which the slope of a regression line is the canonical example. Because $\arctan({\overset{ˆ}{\eta }}_{i}/{\overset{ˆ}{\xi }}_{i})$ depends only on the ratio, it performs this identification automatically and is likewise invariant to the sign indeterminacy of the latent scores; and since β=tanθ maps $\theta \in (-90^{\circ },90^{\circ }]$ bijectively onto R, the angle carries precisely the information the segmentation requires and nothing else.

Replacing arctan by atan2 breaks this identification. With $\beta_{A}=+1$, observations with ${\overset{ˆ}{\xi }}_{i}>0$ map to $+45^{\circ }$ while those with ${\overset{ˆ}{\xi }}_{i}<0$ map to $-135^{\circ }$, so every true subgroup is split into two branches $180^{\circ }$ apart. A two-segment cut of the resulting configuration then pairs one branch of each true subgroup with one branch of the other, and each recovered segment ends up holding an equal mixture of the two directions.

The identification can also be made explicit through the standard axial-data device of angle doubling. Since 2arctan(y/x) and 2atan2(y,x) differ by a multiple of 2π, the embedding z=(cos2θ,sin2θ) is numerically identical under either definition, the largest discrepancy over 5,000 points being $3.8\;\times 10^{-11}$, so the question of quadrant ambiguity is settled by construction. We nevertheless retain the linear θ scale for clustering. The intrinsic axial metric treats $+89^{\circ }$ and $-89^{\circ }$ as neighbours and therefore merges steeply positive with steeply negative relationships. This is immaterial when the latent scores are well determined, only 3% of simulated observations lying beyond $|\theta |>75^{\circ }$, but not in survey data, where that share reaches 27.4% in the real-case application: there the embedding changes the split from 4,188 and 1,135 respondents with coefficients +0.822 and −0.553 to 2,366 and 2,957 with +0.914 and +0.029, losing the sign reversal entirely. On the linear scale θ is a monotone reparameterisation of the signed slope, and the endpoints $\pm 90^{\circ }$ are simply the limits β→±∞, approached only for the low-radius observations that the trimming step already removes.

One consequence of the standardization built into PLS-SEM deserves to be stated explicitly, because it fixes the scale on which the whole criterion operates. Both composite scores are standardised before Eq. (7) is applied, so the slope of the structural relationship inside a segment is a correlation and is confined to [−1,1]. The line of a segment therefore always lies within $\pm 45^{\circ }$ of the $\overset{ˆ}{\xi }$ axis, whatever the magnitude of the coefficient in the population, and the largest angular separation two segments can display is $90^{\circ }$. That maximum is already attained by a symmetric sign reversal β=(+1,−1), which yields local correlations of ±0.996 and a separation of $89.76^{\circ }$; raising the true coefficient to ±3, ±10 or ±50 moves the segment angle only from $44.88^{\circ }$ to $44.97^{\circ }$, because a larger coefficient raises the signal-to-noise ratio rather than the slope. The same arithmetic shows that the separation is governed by reliability and not by the arithmetic difference between the coefficients. A contrast that looks far more modest, β=(+1,−0.3), still produces local correlations of +0.996 and −0.967 and a separation of $88.91^{\circ }$, while the same-sign contrast β=(+0.9,+0.3) produces +0.995 and +0.967 and a separation of only $0.83^{\circ }$. What the angular criterion responds to is the sign structure, not the size of the coefficients.

Two consequences follow, and both matter for reading the results below. First, the criterion is invariant to the scale on which the structural coefficient is expressed, so no rescaling of the constructs can create or destroy detectable directional heterogeneity. Second, because Δθ can never exceed $90^{\circ }$, the operating region derived in the sub-section on $\Delta \theta_{\text{min}}$ is bounded above: a study whose required $\Delta \theta_{\text{min}}$ exceeds that ceiling lies outside the reach of the method however large the sample. Observations close to the $\pm 90^{\circ }$ endpoints likewise do not come from steep coefficients. They come from a small radius $r=\sqrt{{\hat{\xi }}_{i}^{2}+{\hat{\eta }}_{i}^{2}}$, that is from units whose exogenous score is near zero, which becomes common when the indicators are unreliable: the share of observations with $|\theta |>75^{\circ }$ rises from 2.1% at λ=0.9 and σ=0.1 to 10.3% at λ=0.6, σ=0.6 and 12.9% at λ=0.5, σ=0.8, while the corresponding separation falls from $89.76^{\circ }$ to $78.33^{\circ }$ and then to $66.73^{\circ }$. This is the same population that the trimming step of the previous sub-section removes.

(8) $$\begin{array}{c} \theta =\left[\begin{array}{c} \theta_{1}\\ \theta_{2}\\ \vdots \\ \theta_{i}\\ \vdots \\ \theta_{n} \end{array}\right] \end{array}$$

θ obtained from Eq. (8) is then standardized. Hierarchical Agglomerative Clustering (HAC) [28] is then applied to the standardized **θ**, using Euclidean distance and the Ward linkage method to group observations with similar structural angles. The cutoff point of the dendrogram is the largest distance to establish the most suitable numbers of clusters (C) [29]. The PCA-PI-PLS Algorithm (step 1 until 3 in Table 3) is also re-run in each of the clusters to achieve respective path coefficients of every group.

**Table 3:** PCA-PI-PLS-ABS algorithm.

1. Data Preparation and Initialization Stage 1.1. Design an output in the form of a list, but still empty. 1.2. Calculate the number of indicators 1.3. Calculate the number of latent variables 1.4. Calculate the number of observations 1.5. Standardize the data in matrix form. 1.6. Mapping indicators to latent variables 1.7. Mapping the outer model type 1.8. Converting standardization into list form **1.9. Weight of PCA-PI** 1.10. Calculating the f value 1.11. Calculating outer weights 1.12. Outside approximation
2. Steps for Estimating Path Coefficients and Outer Loadings 2.1. Creating a list of exogenous columns 2.2. Creating an exogenous matrix 2.3. Calculating Path Coefficients 2.4. Calculating Outer Loadings 2.5. Calculating R^2^
3. Location parameter estimation stages 3.1. Calculating m 3.2. Calculating nx 3.3. Calculating p_0_ 3.4. Calculating b_0_
**4. ABS Segmentation** **4.1. Calculating**θ **4.2. Standardization**θ **4.3. Segmentation using HAC analysis based on standardization results**θ
**5. Repeat Steps 1 to 3 for each segment/group/cluster formed**

### Criteria for evaluating a segmentation solution

Because a segmentation must be judged against the aggregate model it replaces, three criteria are used throughout. Let K be the number of segments, $n_{k}$ the size of segment k, $n=\sum_{k}n_{k}$ the sample size, and $R_{k}^{2}$ the coefficient of determination of the endogenous construct in the local model of segment k. The sample-size-weighted coefficient of determination and the gain it achieves over the aggregate model are:

(9) $$\begin{array}{c} R_{weighted}^{2}=\sum_{k=1}^{K}\frac{n_{k}}{n}R_{k}^{2},\;\;R_{gain}^{2}=R_{weighted}^{2}-R_{\text{global}}^{2}\; \end{array}$$

Weighting by segment size is what makes the measure honest: without it a method could appear successful by isolating a small pocket of respondents that fits almost perfectly while leaving the bulk of the sample unexplained. Because $R_{weighted}^{2}$ speaks only about the structural model, a segmentation that raised it by damaging the measurement model would still score well. The Group Quality Index [30] closes that gap. Writing $e_{iq}$ for the measurement residual of indicator q on unit i and $f_{i}$ for the structural residual of unit i, both taken under the local model to which unit i is assigned, and $x_{iq}$ and ${\overset{ˆ}{\eta }}_{i}$ for the standardised indicator and endogenous score, the index is,

(10) $$GQI=\sqrt{\left(1-\frac{\sum_{i}\sum_{q}e_{iq}^{2}}{\sum_{i}\sum_{q}x_{iq}^{2}}\right)\left(1-\frac{\sum_{i}f_{i}^{2}}{\sum_{i}{\hat{\eta }}_{i}^{2}}\right)}$$

The first factor is a pooled communality index and the second a pooled redundancy index. Both are computed once over all units rather than averaged over per-segment summaries, so a segmentation cannot improve the index by moving poorly fitting units into a segment whose own statistics then absorb them. For K=1 the expression reduces exactly to the goodness-of-fit of the aggregate model, which gives every comparison a natural benchmark: a segmentation is worth reporting only if its GQI exceeds that value. Each method is allowed to choose its own number of segments under its own rule, so that K is a result rather than an assumption.

### Acceptance protocol for a reported segmentation

A high $\Delta R^{2}$ is not by itself evidence of heterogeneity, because the same quantity can be produced by a clustering algorithm that has merely fitted noise in a single homogeneous population. Three diagnostics are therefore attached to every segmentation reported in this paper. They answer three different questions: could the gain have arisen without heterogeneity, does the gain survive on units that did not shape the partition, and are the same units grouped together when the sample changes.

The first diagnostic is a homogeneous residual bootstrap on the $R_{weighted}^{2}$ gain. The natural-looking alternative, a permutation test that randomly reorders $\overset{ˆ}{\eta }$, tests the null of no relationship at all; it destroys the aggregate structural path as well as the heterogeneity, so it is far too easy to reject and is not the hypothesis at issue. The relevant null is that a single homogeneous population generated the data with the aggregate coefficient $\overset{ˆ}{\beta }=\text{cor}\left(\overset{ˆ}{\xi },\overset{ˆ}{\eta }\right)$. Writing ${\overset{ˆ}{e}}_{i}={\overset{ˆ}{\eta }}_{i}-\overset{ˆ}{\beta }{\overset{ˆ}{\xi }}_{i}$ for the structural residuals and $\pi_{b}$ for an independent random permutation of {1,…,n}, synthetic samples are generated as,

$$\begin{array}{c} \eta_{i}^{*\left(b\right)}=\overset{ˆ}{\beta }\;{\overset{ˆ}{\xi }}_{i}+{\overset{ˆ}{e}}_{\pi_{b}\left(i\right)},\;\;i=1,\ldots ,n,\;b=1,\ldots ,B \end{array}$$

each standardised and then passed through the complete segmentation pipeline, exactly as the observed data were, giving a null value $\Delta R^{2(b)}$ per draw. Reassigning the residuals by permutation preserves their empirical distribution exactly, including any heavy tails, while removing every association between a unit and its own residual, so a synthetic sample has one structural coefficient by construction and any gain it shows is the algorithm's own capacity to over-fit. The p-value is,

$$p=\frac{1+\#\left\{b:\Delta R^{2\left(b\right)}\geq \Delta R_{\text{obs}}^{2}\right\}}{B+1},\;\;B=100\;\text{or}\;200$$

The second diagnostic asks whether the gain is real out of sample. Because $R_{weighted}^{2}$ is computed on the same units that produced the partition, it is optimistic by construction. For each of S=50 random splits the sample is divided into halves $T_{b}$ and $V_{b}$; the segmentation is estimated on $T_{b}$ alone, yielding centroids $c_{k}^{\left(b\right)}$ in the angular feature space; every validation unit is then assigned by the nearest-centroid rule ${\overset{ˆ}{k}}_{i}=\arg\min_{k}\left|z_{i}-c_{k}^{\left(b\right)}\right|$ without using its own ${\overset{ˆ}{\eta }}_{i}$ in the assignment, and the $R_{weighted}^{2}$ is recomputed on $V_{b}$ only:

$$R_{weighted,\text{out}}^{2}=\frac{1}{S}\sum_{b=1}^{S}\sum_{k=1}^{K}\frac{\left|V_{bk}\right|}{\left|V_{b}\right|}\;r^{2}\left(\overset{ˆ}{\xi },\overset{ˆ}{\eta };V_{bk}\right),\;\;V_{bk}=\left\{i\in V_{b}:{\overset{ˆ}{k}}_{i}=k\right\}$$

A partition that reflects a genuine mechanism reproduces itself on units it never saw, so $R_{weighted,\text{out}}^{2}\approx R_{weighted}^{2}$; a partition that has fitted noise collapses towards the aggregate $R^{2}$. The third diagnostic asks whether the partition itself, and not merely its explanatory power, is reproducible. Let ${\overset{ˆ}{C}}_{1},\ldots ,{\overset{ˆ}{C}}_{K}$ be the reference segments on the full sample. For b=1,…,S a subsample $U_{b}$ of ⌊n/2⌋ units is drawn without replacement, the whole segmentation is re-estimated on $U_{b}$ giving $C_{1}^{*(b)},\ldots ,C_{K}^{*(b)}$, and each reference segment is matched to its best-overlapping recovered segment:

$$J_{k}=\frac{1}{S}\sum_{b=1}^{S}\max_{j}\frac{\left|\left({\overset{ˆ}{C}}_{k}\cap U_{b}\right)\cap C_{j}^{*\left(b\right)}\right|}{\left|\left({\overset{ˆ}{C}}_{k}\cap U_{b}\right)\cup C_{j}^{*\left(b\right)}\right|}$$

Subsampling without replacement is essential here. Under the ordinary nonparametric bootstrap only $1-e^{-1}\approx 63.2\%$ of the units appear in a draw, so the Jaccard index is mechanically bounded near 0.63 even for a perfectly stable partition and the diagnostic becomes uninformative. Segments are indexed by the sign of their local coefficient so that $J_{1}$ refers to the positive and $J_{2}$ to the negative segment. Following [31] a value of $J_{k}\geq 0.75$ is taken as evidence of a stable segment. A segmentation is reported in this paper only if all four conditions hold: $\Delta R^{2}>0$, p<0.05, $R_{weighted,\text{out}}^{2}$ close to $R_{weighted}^{2}$, and $J_{k}\geq 0.75$ for every segment. The first condition is not redundant. The bootstrap compares the observed gain with what the same algorithm produces under homogeneity, so a procedure that systematically damages the model can still yield a small p-value while its $R_{weighted}^{2}$ lies below the aggregate $R^{2}$.

## Method validation

### Initial simulation

To validate the accuracy of this method, a Monte Carlo simulation was used with the R programming language. The simulation was conducted by generating two data groups, group A with 100 observations and group B with 100 observations. Data generation began by defining exogenous latent variables (ξ) for both groups, where$\;\xi_{A}$ and $\xi_{B}$ were normally distributed:

$$\xi_{A},\xi_{B}∼N\left(0,1.5^{2}\right)$$

The exogenous latent variables (ξ ) for both groups were randomly generated following a normal distribution with expanded variance ($\sigma^{2}=1.5^{2}$) to emphasize the geometric pattern of the data.

Furthermore, the endogenous latent variables (η) are arranged as a linear function of ξ with diametrically opposite directions of relationship. For Group A, the path coefficient is set to perfectly positive ($\beta_{A}=1.0$), while for Group B it is set to perfectly negative ($\beta_{B}=-1.0$). Mathematically, the structural equation (inner model) for both groups can be written as follows:

$$\eta_{A}=\xi_{A}+\zeta_{A}$$

$$\eta_{B}=-\xi_{B}+\zeta_{B}$$

Where $\zeta_{A},\zeta_{B}∼N\left(0,0.1^{2}\right)$ is the disturbance term representing minor variations.

After generating latent scores from exogenous and endogenous latent variables, the indicators for each variable are then generated. In this simulation, three indicator generations were performed for each latent variable, starting from x_1_, x_2_ and x_3_ for exogenous variables until y_1_, y_2_ and y_3_ for endogenous variables. This was done in all groups, both A and B. All of them used reflective measurements. To ensure high convergent validity and minimize measurement bias, the factor loading (λ) was set uniformly at 0.90. Each indicator contained a random measurement error component that was normally distributed $N(0,0.1^{2})$ . The measurement equation was defined as follows:

$$x_{hA}=0.95\cdot \xi_{A}+\delta_{hA}$$

$$x_{hB}=0.95\cdot \xi_{B}+\delta_{hB}$$

$$y_{hA}=0.95\cdot \eta_{A}+\varepsilon_{hA}$$

$$y_{hB}=0.95\cdot \eta_{B}+\varepsilon_{hB}$$

Where h=1,2,3 , and $\delta ,\varepsilon ∼N(0,0.1^{2})$ represent measurement errors. The head and tail data from indicators and grouping labels can be seen in Table 4.

**Table 4:** Simulation data and original grouping.

No	x_1_	x_2_	x_3_	y_1_	y_2_	y_3_	Group
1	1445	1469	1470	1371	1430	1370	A
2	0.97	0.892	0.806	0.837	0.959	0.849	A
⁝	⁝	⁝	⁝	⁝	⁝	⁝	⁝
199	−0.879	−0.848	−0.793	0.785	0.901	0.722	B
200	0.758	0.589	0.672	−0.971	−0.626	−0.788	B

From the data generated in Table 4, a global PCA-PI-PLS analysis was performed (groups A and B were combined into one). The structural model used in this simulation can be seen in Fig. 1. After conducting a global PCA-PI-PLS analysis, the path coefficient value obtained for the influence of ξ on η was 0.013 (this influence value is very small because it is close to zero), as can be seen in Fig. 2. However, does this value truly represent the actual influence pattern? To identify this, it is necessary to look at the plot of the latent scores of the exogenous and endogenous variables formed (see Fig. 3).

**Fig. 1:**
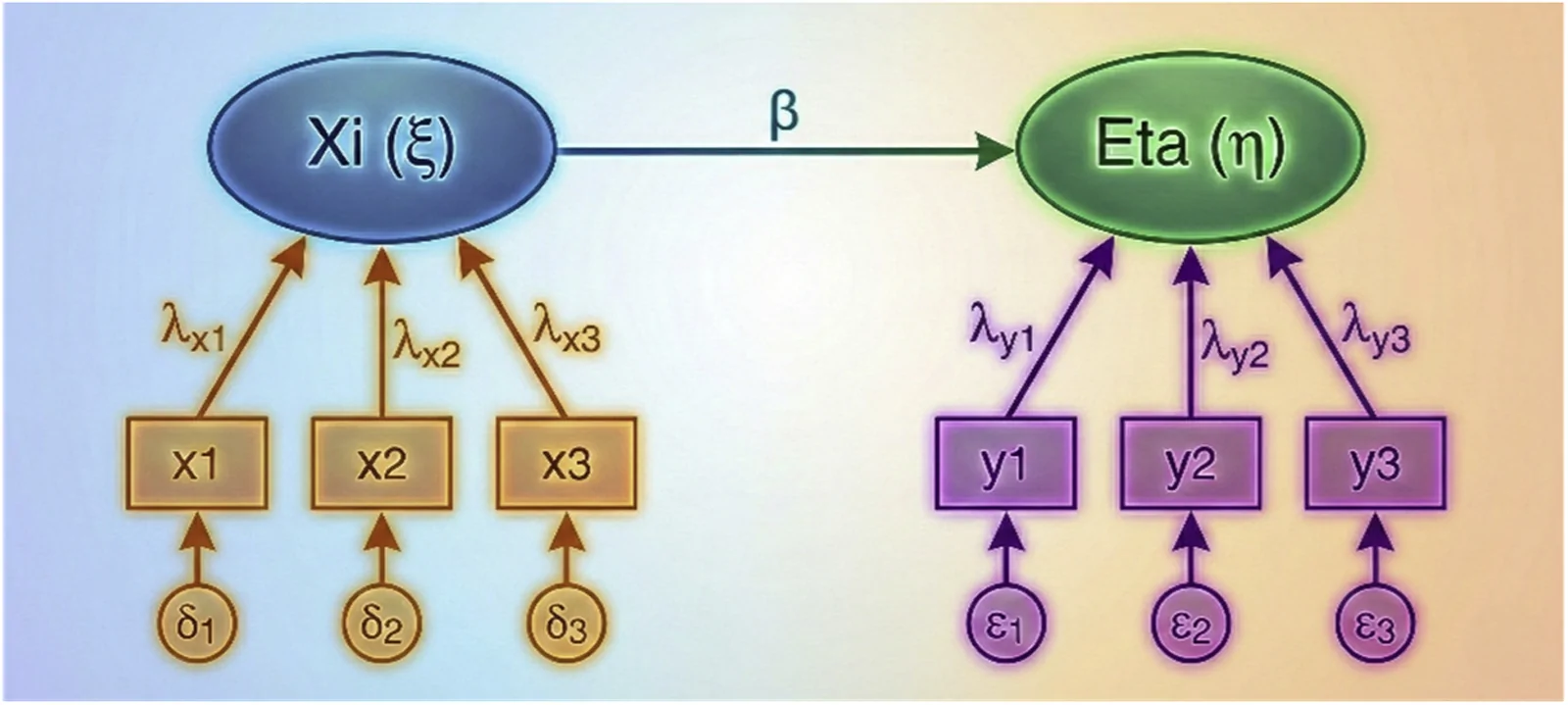
Structural model design in simulation.

**Fig. 2:**
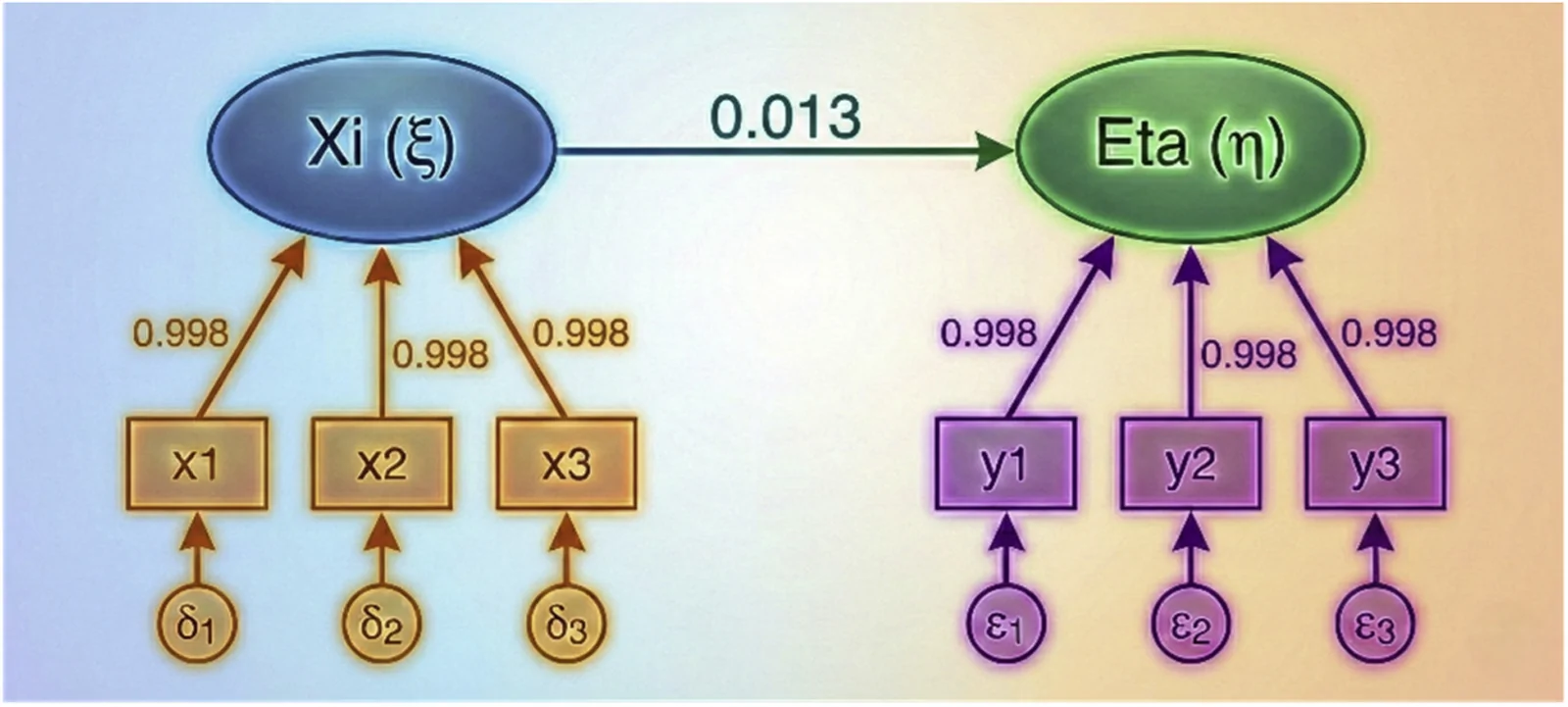
Global PCA-PI-PLS results.

**Fig. 3:**
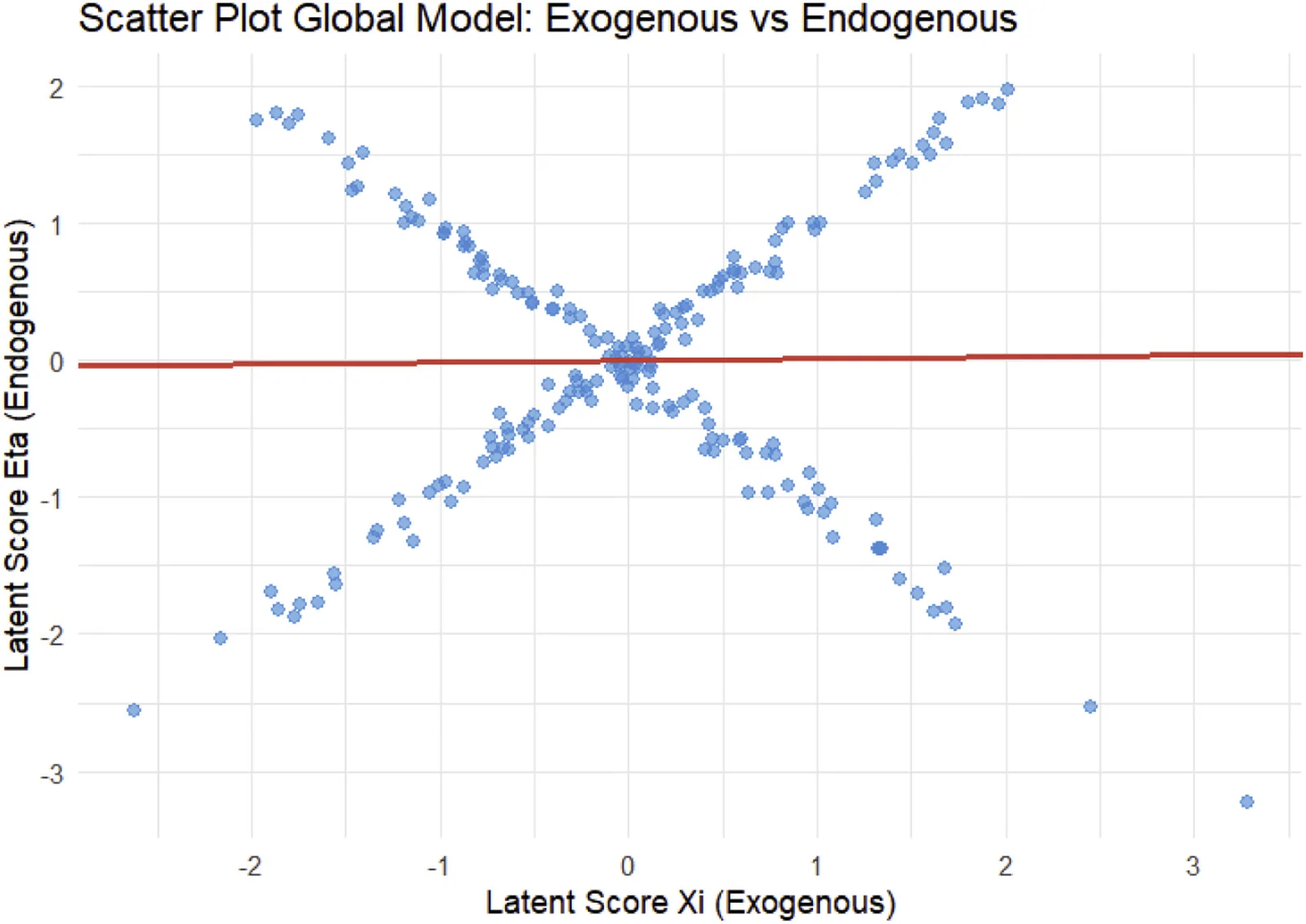
Scatter plot of exogenous latent scores vs. endogenous latent scores in the global model.

Based on Fig. 3, it can be seen that the data should form two straight lines, where one line shows that the higher the exogenous latent score, the higher the endogenous latent score. Conversely, for the other line, the higher the exogenous latent score, the lower the endogenous latent score. However, in reality, the regression line formed using PCA-PI-PLS globally produces a flat line. The solution to this problem requires segmentation that divides the data into two groups, where group A follows the ascending line, while group B follows the descending line. Using the *Angle Based Segmentation (ABS)* proposed in this study, first, a transformation of the θ is required based on equations 14 and 15. The results of the transformation are then standardized, and a *Hierarchical Agglomerative Clustering (HAC)* analysis is performed based on the standardized θ. The results of this *Angle Based Segmentation (ABS)* can be seen in the dendrogram in Fig. 4.

**Fig. 4:**
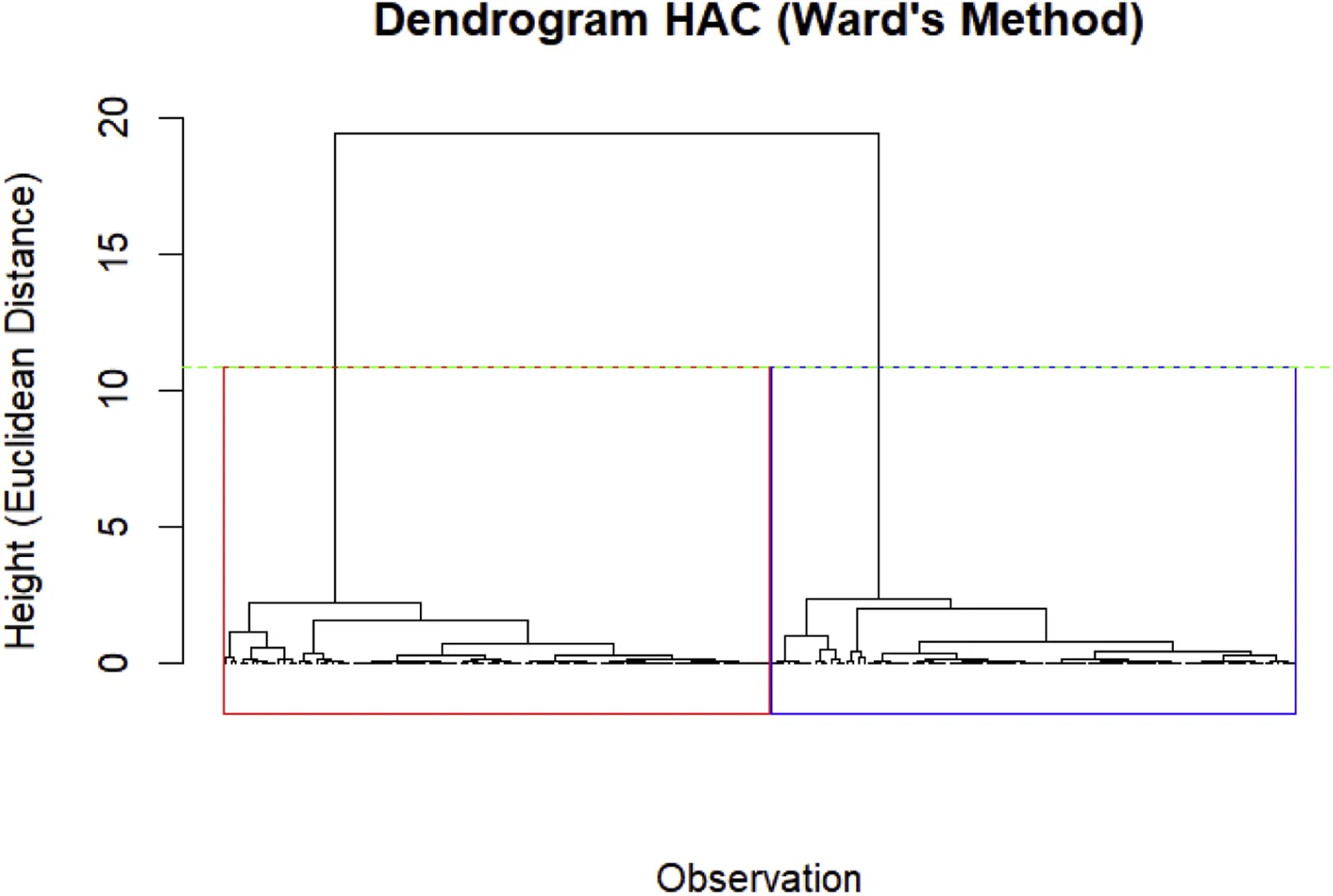
Hierarchical agglomerative clustering (HAC) dendrogram using standardized θ**.**

According to Fig. 4, it can be seen that the largest distance difference occurred during the formation of two groups, so from the dendrogram, the best segmentation is to divide it into two groups, namely A (Red) and B (Blue). The results of grouping with *Angle Based Segmentation (ABS)* can be seen in Table 5.

**Table 5:** Simulation data and clustering results of angle-based segmentation (ABS).

No	x_1_	x_2_	x_3_	y_1_	y_2_	y_3_	ABS Group
1	1445	1469	1470	1371	1430	1370	A
2	0.97	0.892	0.806	0.837	0.959	0.849	A
⁝	⁝	⁝	⁝	⁝	⁝	⁝	⁝
199	−0.879	−0.848	−0.793	0.785	0.901	0.722	B
200	0.758	0.589	0.672	−0.971	−0.626	−0.788	B

Performance validation of the segmentation was conducted by comparing the prediction results of *Angle Based Segmentation (ABS)* (Table 5) with the original labels of the simulation dataset (Table 4), as summarized in Table 6 in the form of a Contingency Table. In the contingency table, the number of members of group A who were correctly assigned to group A, members of group A who were incorrectly assigned to group B, members of group B who were correctly assigned to group B, and members of group B who were incorrectly assigned to group A were calculated.

**Table 6:** Contingency table real group Vs ABS segmentation result.

		Predicted (ABS Segmentation)	
		A	B
Real Group	A	97	3
	B	5	95

The contingency table in Table 6 shows a very strong level of concordance, marked by the concentration of data on the diagonal of the matrix. Based on Table 6, the accuracy of the grouping can be measured as follows [32]:

(11) $$\begin{array}{c} Accuracy=\frac{Sum\;of\;Diagonal\;Contingency\;Table}{Sum\;of\;Contingency\;Table}=\frac{97+95}{97+3+5+95}=0.96=96\% \end{array}$$

The overall accuracy calculation, as formulated in Eq. (11), yields a value of 96%. Under the simulated conditions the angular transformation recovers the two opposing subgroups with a balanced accuracy of approximately 95%. The conditions under which this figure transfers are examined in the sub-sections on robustness and on the operating region. The ABS algorithm in this simulation has been proven to successfully capture structural information that is usually lost in global analysis due to unobserved heterogeneity in the data.

In addition to clustering accuracy, the path coefficient (β) and the level of explained variance ($R^{2}$) from the PCA-PI-PLS results of each group were also measured. The $R^{2}$ values of each group were compared with the global $R^{2}$. The results of this comparison can be seen in Table 7.

**Table 7:** PCA-PI-PLS (global) vs. PCA-PI-PLS-ABS (local/segmentation).

Simulation	β			$R^{2}$		
	Global	Group A	Group B	Global	Group A	Group B
1	0.0133	0.9955	−0.9945	0.0002	0.9910	0.9890

The impact of ignoring unobserved heterogeneity is clearly seen in Table 7. The global PCA-PI-PLS analysis failed to capture the existing phenomenon, as shown in Fig. 3 and the value of ${R^{2}}_{Global}$ in Table 7 of 0.0002. The global model only captured the average of the two opposing forces, resulting in an almost flat regression line (β≈0 ). The application of the ABS segmentation method successfully solved this problem by separating observations based on their geometric orientation. As a result, the segmented model was able to reveal the 'mask' of heterogeneity. In Group A, a very strong positive path was identified (β=0.9955), while in Group B, an equally strong negative path was identified (β=−0.9945). In addition, the increase in the $R^{2}$ was also massive, from 0.02% to 99.1% in group A and 98.9% in group B. This proves that the local model provides a much more accurate and valid representation of the data than the global model.

A visual comparison between the global model and the local model provides an illustration of what a global model can conceal when directional heterogeneity is present in the data. Fig. 3 shows a scatter plot of the Global Model estimation results. In this graph, the data points form a cross pattern resembling the letter "X", which indicates two opposing trends in a single dataset. However, because the PCA-PI-PLS (Global) analysis forces a single regression line for all data, the algorithm is only able to capture the average value of the two trends. As a result, the resulting regression line (red line in Fig. 3) appears flat or horizontal with a slope close to zero. Visually and statistically, this gives the misleading conclusion that there is no relationship between the exogenous and endogenous variables, when in fact there is a very strong but canceling effect. This contradiction is completely resolved in Fig. 5, which shows the estimation results after applying the *Angle Based Segmentation (ABS)* method. The left graph in Fig. 5 shows that the algorithm successfully isolated the data group with a positive trend. The regression line rises sharply with a path coefficient of 0.995, and the data points cluster very closely along the line, indicating an almost perfect model fit. In contrast, the graph on the right shows the data group with a negative trend. The regression line declines sharply with a coefficient of −0.994, which is also followed by high prediction accuracy. The transformation from Figs. 3, 4, and 5 illustrates the success of the proposed method in handling the Simpson's Paradox phenomenon. This method not only restores the true direction of the relationship (positive and negative), but also restores the predictive power that was previously lost due to bias in the global model.

**Fig. 5:**
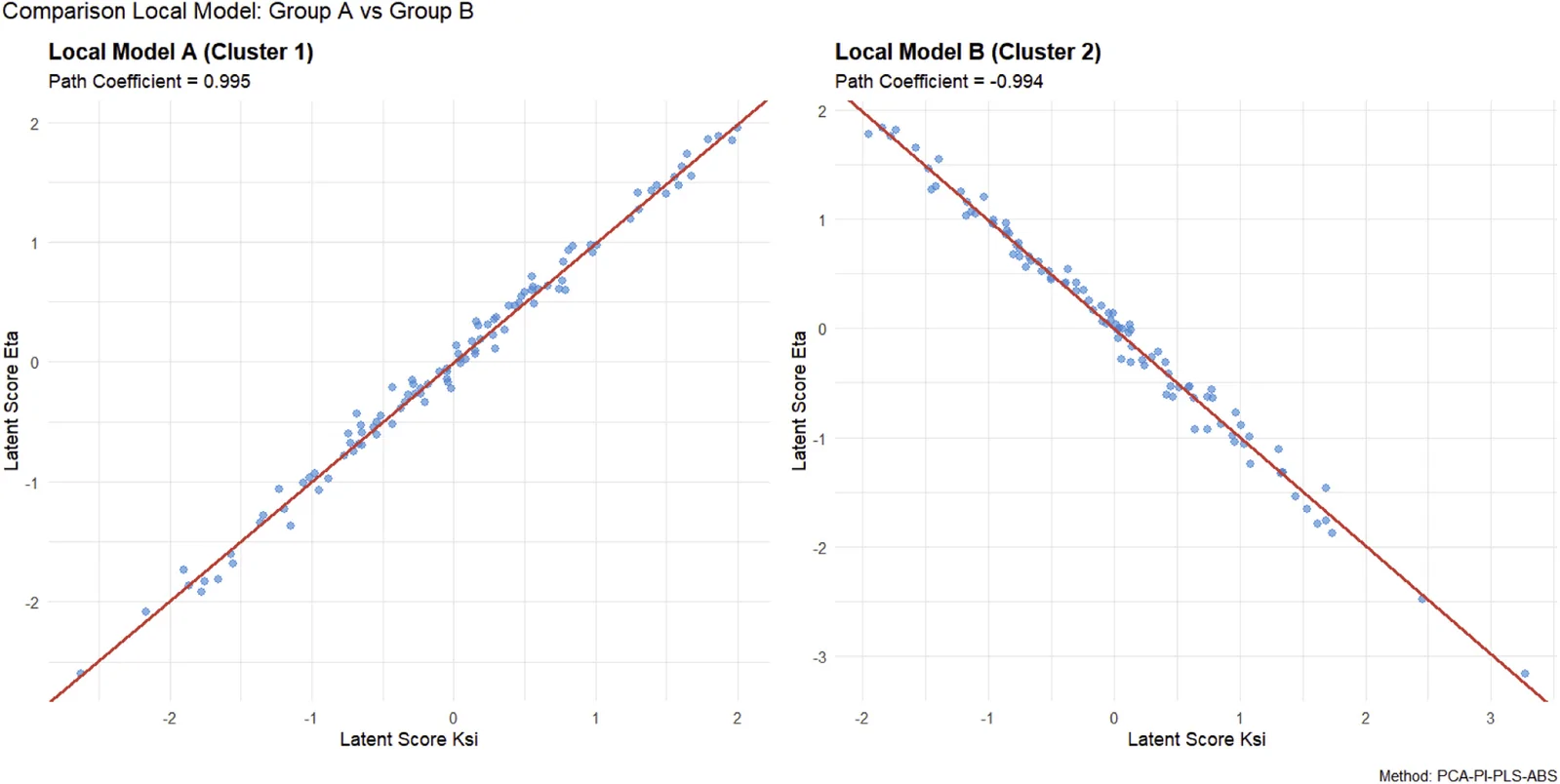
Scatter plot of exogenous latent scores vs. endogenous latent scores in local models.

### Simulation repeats with different data but the same path coefficient

To confirm that the proposed method is valid and reliable for handling cases with different data but with extreme variations in the direction of the relationship, the simulation was repeated for different data 10 times (by changing the set.seed value in R) but with the same number of samples ($n_{A}=100$ , and $n_{B}=100$) and the same initial path coefficient settings ($\beta_{A}=1.0$ , and $\beta_{B}=-1.0$). The results of the simulation repetitions can be seen in Table 8.

**Table 8:** Comparison of results for different data but same path coefficients ($\beta_{A}=1.0$ and $\beta_{B}=-1.0$).

Simulation	Setting Set Seed in R	Accuracy	β			$R^{2}$		
			Global	Group A	Group B	Global	Group A	Group B
1	771	96%	0.0133	0.9955	−0.9945	0.0002	0.9910	0.9890
2	772	94	0.0020	0.9946	−0.9961	3.9 × 10^−6^	0.9892	0.9922
3	773	94.5%	0.0261	0.9960	−0.9962	0.0007	0.9921	0.9925
4	774	97.5%	0.0841	0.9973	−0.9970	0.0071	0.9946	0.9939
5	775	96%	0.0362	0.9954	−0.9957	0.0013	0.9908	0.9913
6	776	95	0.0512	0.9957	−0.9950	0.0026	0.9913	0.9901
7	777	95.5%	0.0632	0.9955	−0.9948	0.0040	0.9909	0.9896
8	778	95%	0.0273	0.9943	−0.9954	0.0007	0.9887	0.9908
9	779	96.5%	−0.0118	0.9960	−0.9964	0.0001	0.9920	0.9928
10	780	95	0.0368	0.9961	−0.9951	0.0014	0.9922	0.9902

Table 8 provides an empirical overview of the robustness of the proposed method in dealing with other random data variations. Although the seed value was changed 10 times to generate different data variations, the resulting conclusion pattern remained identical. The Simpson's Paradox phenomenon was clearly evident in all scenarios. Global analysis always produces path coefficients close to 0 (for example, in Simulation 2, the global $R^{2}$ falls to $3.9\;\times 10^{-6}$. Where directional heterogeneity is present, an aggregate model may report a coefficient close to zero even though strong relationships of opposite sign exist within the sample. Whether such heterogeneity is present in a given dataset is an empirical question, and this article supplies both a procedure for detecting it and a test for deciding whether the detected structure is genuine. However, with the application of ABS, this unobserved heterogeneity is consistently revealed. The algorithm not only successfully separates positive and negative relationships with high precision, but also maintains the explained variance ($R^{2}$) above 98% in each group across all experiments. The consistency of accuracy within the range of 94%−97.5% confirms that the geometric angle transformation with HAC grouping, named *Angle Based Segmentation (ABS)*, performs consistently within the operating region characterised by the expression for $\Delta \theta_{\text{min}}$.

### Simulation iterations with different path coefficients

Further validation was conducted to test how extreme the angle differences needed to be for Angle Based Segmentation (ABS) to work effectively. To see how extreme the direction differences that could be captured using Angle Based Segmentation (ABS) were, simulations were conducted with the same number of samples ($n_{A}=100$ and $n_{B}=100$) and the initial path coefficient settings for group A remained the same ($\beta_{A}=1.0$). The only difference was the initial path coefficient for group B ($\beta_{B}$) with experiments at −1.0, −0.75, −0.50, −0.25, 0, 0.25, 0.50 and 0.75. The value 1 was not used for$\beta_{B}$ because it would be the same as the value$\beta_{A}$. The results of the simulation can be seen in Table 9.

**Table 9:** Comparison of results for different path coefficients.

Simulation	$\beta_{A}$	$\beta_{B}$	Accuracy	β			$R^{2}$		
				Global	Group A	Group B	Global	Group A	Group B
1	1.0	−1.0	96	0.0133	0.9955	−0.9945	0.0002	0.9910	0.9890
11	1.0	−0.75	95.5%	0.1530	0.9955	−0.9916	0.0234	0.9911	0.9832
12	1.0	−0.50	92.5%	0.3246	0.9953	−0.9846	0.1054	0.9907	0.9694
13	1.0	−0.25	92.5%	0.5181	0.9955	−0.9488	0.2684	0.9910	0.9003
14	1.0	0	92%	0.7056	0.9955	−0.0333	0.4980	0.9912	0.0011
15	1.0	0.25	88	0.8528	0.9964	0.9488	0.7273	0.9930	0.9003
16	1.0	0.50	50.5%	0.9432	0.9438	−0.7904	0.8896	0.8908	0.6248
17	1.0	0.75	50.5%	0.9849	0.9855	−0.7542	0.9701	0.9711	0.5689

By maintaining the $\beta_{A}$ at 1.0 and varying the $\beta_{B}$ from −1.0 to 0.75, the simulations in Table 9 reveal the geometric characteristics of this method. The results show a strong positive correlation between the magnitude of the vector angle difference and segmentation accuracy. In the range where the direction of the relationship is opposite (contrast) or orthogonal ($\beta_{B}$ from negative to 0), the algorithm is able to separate data with high precision above 92%, proving its reliability in handling Simpson's Paradox cases and nil relationships. However, Table 9 also shows the "saturation point" of this angle-based approach. When the $\beta_{B}$ shift to a strong positive area (0.50 and 0.75), the latent score vectors of the two groups become almost parallel (collinear), causing the angle difference (Δθ) to become too small to detect. As a result, accuracy falls to 50.5%, which is equivalent to random chance. This finding confirms that the ABS method is a very powerful specific solution for detecting directional heterogeneity (positive vs. negative) or significant differences in magnitude, but is not designed to distinguish subtle variations in intensity within the same direction.

### Real-case application

To demonstrate that the algorithm is informative outside a controlled environment it was applied to an empirical survey of 5,323 respondents measuring the effect of Teacher Achievements on the Quality of Teacher Education, each construct being measured reflectively by five items on a five-point Likert scale. The $R^{2}$ of Global PCA-PI-PLS model is 0.3834 and the Group Quality Index (GQI) is 0.5185 (See Table 10). All three segmentation methods were applied to this data, with each method determining its own number of segments.

**Table 10:** Real-case application: comparison of the three segmentation methods.

Model	K detected	$R_{weighted}^{2}$	GQI	R² gain
Global (no segmentation)	1	0.3834	0.5185	0
PCA-PI-PLS-ABS (proposed)	2	0.5966	0.6425	0.2132
FIMIX-PLS	1	0.3834	0.5185	0
REBUS-PLS	3	0.4307	0.5381	0.0473

Table 10 shows that the three methods reach different conclusions about the same sample. FIMIX-PLS retains K=1: although the likelihood improves with K, no multi-class solution is admissible, because the normed entropy is 0.303 at K=2, 0.445 at K=3 and 0.453 at K=4, all below the 0.50 separability threshold. REBUS-PLS retains K=3 but achieves only $R_{gain}^{2}=0.0473$ and a GQI of 0.538. PCA-PI-PLS-ABS retains K=2 using the farthest distance cutoff approach. The weighted coefficient of determination rises from 0.3834 to 0.5966, a gain of $R_{gain}^{2}=0.2132$, and the GQI rises from 0.5185 to 0.6425, so this increase is the largest among the three segmentation methods.

A real sample carries no ground truth, so the reality of the split must be tested rather than assumed, and the null hypothesis has to be chosen with care. The relevant null is not that the constructs are unrelated but that the sample comes from a single homogeneous population whose path coefficient equals the aggregate coefficient. We therefore use a residual bootstrap under homogeneity: holding $\overset{ˆ}{\xi }$ fixed, we generate $\eta^{*}={\overset{ˆ}{\beta }}_{\text{global}}\overset{ˆ}{\xi }+\varepsilon^{*}$ with $\varepsilon^{*}$ resampled from the residuals of the aggregate model, repeat the whole segmentation on each replicate, and take $R_{gain}^{2}$ as the test statistic. The partition was also re-estimated out of sample over 50 random half-splits with a nearest-centroid assignment rule, and its stability was measured by the half-sample Jaccard index.

Table 11 shows that only two of the five quantities commonly reported are decisive. The observed gain of 0.2132 lies outside the entire null distribution, whose maximum over 200 replicates is 0.1454, giving p=0.005; and the gain replicates out of sample, with a $R_{weighted}^{2}$ of 0.5959 against 0.5966 in sample and local coefficients of +0.8204 and −0.5569. The remaining quantities do not discriminate. Under the homogeneous null a stable two-segment partition with opposite local slopes arises routinely: the mean angular separation is already $78.4^{\circ }$ against the observed $70.3^{\circ }$ (p=0.775), and the mean lower local coefficient is already −0.5193 against the observed −0.5529 (p=0.603), while the Jaccard index is high under the null as well. Neither a sign reversal nor an angular separation nor a stability index may therefore be reported as evidence in its own right. One boundary should be stated alongside this positive result: with a within-segment angular dispersion of $\sigma_{\theta }=21.8^{\circ }$ and a smaller segment of 1,135 respondents, the feasibility criterion requires a separation of about $112^{\circ }$ against the $70.3^{\circ }$ available, so the assignment of individual respondents should not be treated as a reliable classification. What the analysis establishes, because it survives out-of-sample validation, is the existence, the direction and the approximate magnitude of two opposing structural relationships.

**Table 11:** Real-case application: comparison of the three segmentation methods.

Criterion	Observed	Under homogeneity	p-value	Verdict
Homogeneous residual bootstrap on the $R_{gain}^{2}$	0.2132	max 0.1454	0.0050	decisive, PASS
Out-of-sample $R_{weighted}^{2}$ (50 splits)	0.5959	in-sample 0.5966	-	decisive, PASS
Half-sample Jaccard index (segment 1 / 2)	1.000 / 1.000	threshold 0.75	-	secondary, PASS
Angular separation between segments	70.30°	mean 78.4°	0.775	no discriminating power
Lower local path coefficient	−0.5529	mean −0.5193	0.603	no discriminating power

### Simulation comparison PCA-PI-PLS-ABS with FIMIX-PLS and REBUS-PLS

The simulation design was used to benchmark PCA-PI-PLS-ABS against the two established response-based segmentation techniques in PLS-SEM. FIMIX-PLS was implemented as a finite mixture of regressions on the inner model, estimated by EM, with K chosen by the consistent AIC among admissible solutions, that is those whose normed entropy exceeds 0.50 and whose smallest segment contains at least ten times the number of indicators in the largest block. REBUS-PLS was implemented as the closeness-measure procedure initialised from a hierarchical clustering of the residuals of the aggregate model, with K taken from the farthest-distance rule on that dendrogram. Both were re-implemented in base R so that the comparison isolates the segmentation logic.

Three scenarios were run with 50 replications each at n=200, chosen to separate the kinds of unobserved heterogeneity a segmentation method may face. In S1 the two subgroups have structural relationships of opposite sign, $\beta_{A}=+1$ and $\beta_{B}=-1$. We call this DIRECTIONAL heterogeneity, because the subgroups differ in the direction of the effect and not merely in its size; it is the configuration that produces Simpson's Paradox, since the two effects cancel and the aggregate coefficient is close to zero and describes neither subgroup. S2 keeps the same structural design but replaces the normal structural disturbance by a Student-t distribution with two degrees of freedom, $\zeta ∼t_{2}$, whose heavy tails produce occasional extreme deviations from the subgroup line. This reproduces a familiar feature of survey data and, more importantly, violates the conditional-normal likelihood on which FIMIX-PLS rests, so it tests how far each method depends on that assumption. In S3 the two subgroups share the same direction and differ only in the size of the effect, $\beta_{A}=+0.9$ and $\beta_{B}=+0.3$, both positive. We call this magnitude-only heterogeneity: the effects do not cancel, the aggregate model already fits reasonably well, and the configuration lies outside the scope of the proposed method while being precisely the case for which FIMIX-PLS and REBUS-PLS were designed.

Table 12 and Fig. 6 separates the three methods along the dimension each is designed for. The column K detected reports the modal number of segments retained across the replications, with the percentage of replications in which that number was retained given in brackets, so that a low percentage signals an unstable selection rule. Under directional heterogeneity both PCA-PI-PLS-ABS and FIMIX-PLS retain K=2 in every replication and recover the subgroups almost equally well, with balanced accuracies of 0.954 and 0.958 and gains of $R_{gain}^{2}=0.984$ against an aggregate (global) $R^{2}$ of only 0.007. The proposed method reaches this in 0.003 s against 0.839 s, roughly two orders of magnitude faster, and does so deterministically rather than through repeated random restarts. Once the normality assumption of the mixture likelihood is violated the ordering changes: with heavy-tailed disturbances PCA-PI-PLS-ABS attains an accuracy of 0.904 and a GQI of 0.926 against 0.877 and 0.913 for FIMIX-PLS. When the subgroups differ only in the size of the effect, all three methods stay close to the aggregate model, with gains of at most 0.033, and FIMIX-PLS retains K=1 in 98% of replications, which is exactly what its selection rule should do when no directional structure is present. REBUS-PLS is the least stable of the three: its modal K is 2 but that value is retained in only 44 to 44% of replications, and its accuracy stays near 0.62, because its closeness measure groups units by how well the reflective blocks fit them rather than by the structural mechanism. The three techniques are therefore complementary: the proposed method is the appropriate choice when the direction of the relationship is suspected to vary, and it should not be used when the heterogeneity is expected to lie in effect size alone.

**Table 12:** Simulation benchmark of the three segmentation methods (50 replications per scenario, n = 200).

Scenario	Model	Modus K detected (Percentage)	Mean				
			Accuracy	$R_{weighted}^{2}$	GQI	Time (s)	R² gain
S1. Directional: beta = (+1, −1), opposite signs	Global (no segmentation)	1 (100%)	0.5	0.0072	0.1034	0.001	0
S1. Directional: beta = (+1, −1), opposite signs	PCA-PI-PLS-ABS (proposed)	2 (100%)	0.9535	0.991	0.9937	0.003	0.9838
S1. Directional: beta = (+1, −1), opposite signs	FIMIX-PLS	2 (100%)	0.9581	0.9915	0.994	0.839	0.9843
S1. Directional: beta = (+1, −1), opposite signs	REBUS-PLS	2 (40%)	0.6148	0.2647	0.4028	0.008	0.2575
S2. Directional with heavy-tailed disturbances: beta = (+1, −1), zeta ∼ t(2)	Global (no segmentation)	1 (100%)	0.5	0.0087	0.1098	0.001	0
S2. Directional with heavy-tailed disturbances: beta = (+1, −1), zeta ∼ t(2)	PCA-PI-PLS-ABS (proposed)	2 (100%)	0.904	0.8613	0.9261	0.002	0.8526
S2. Directional with heavy-tailed disturbances: beta = (+1, −1), zeta ∼ t(2)	FIMIX-PLS	2 (96%)	0.8765	0.85	0.9126	0.467	0.8413
S2. Directional with heavy-tailed disturbances: beta = (+1, −1), zeta ∼ t(2)	REBUS-PLS	2 (42%)	0.617	0.2995	0.4592	0.007	0.2908
S3. Magnitude-only: beta = (+0.9, +0.3), same sign	Global (no segmentation)	1 (100%)	0.5	0.6363	0.7965	0.001	0
S3. Magnitude-only: beta = (+0.9, +0.3), same sign	PCA-PI-PLS-ABS (proposed)	2 (92%)	0.5689	0.6696	0.8166	0.001	0.0332
S3. Magnitude-only: beta = (+0.9, +0.3), same sign	FIMIX-PLS	1 (98%)	0.5	0.636	0.7963	1.009	−3e-04
S3. Magnitude-only: beta = (+0.9, +0.3), same sign	REBUS-PLS	2 (44%)	0.5349	0.6456	0.804	0.01	0.0093

**Fig. 6:**
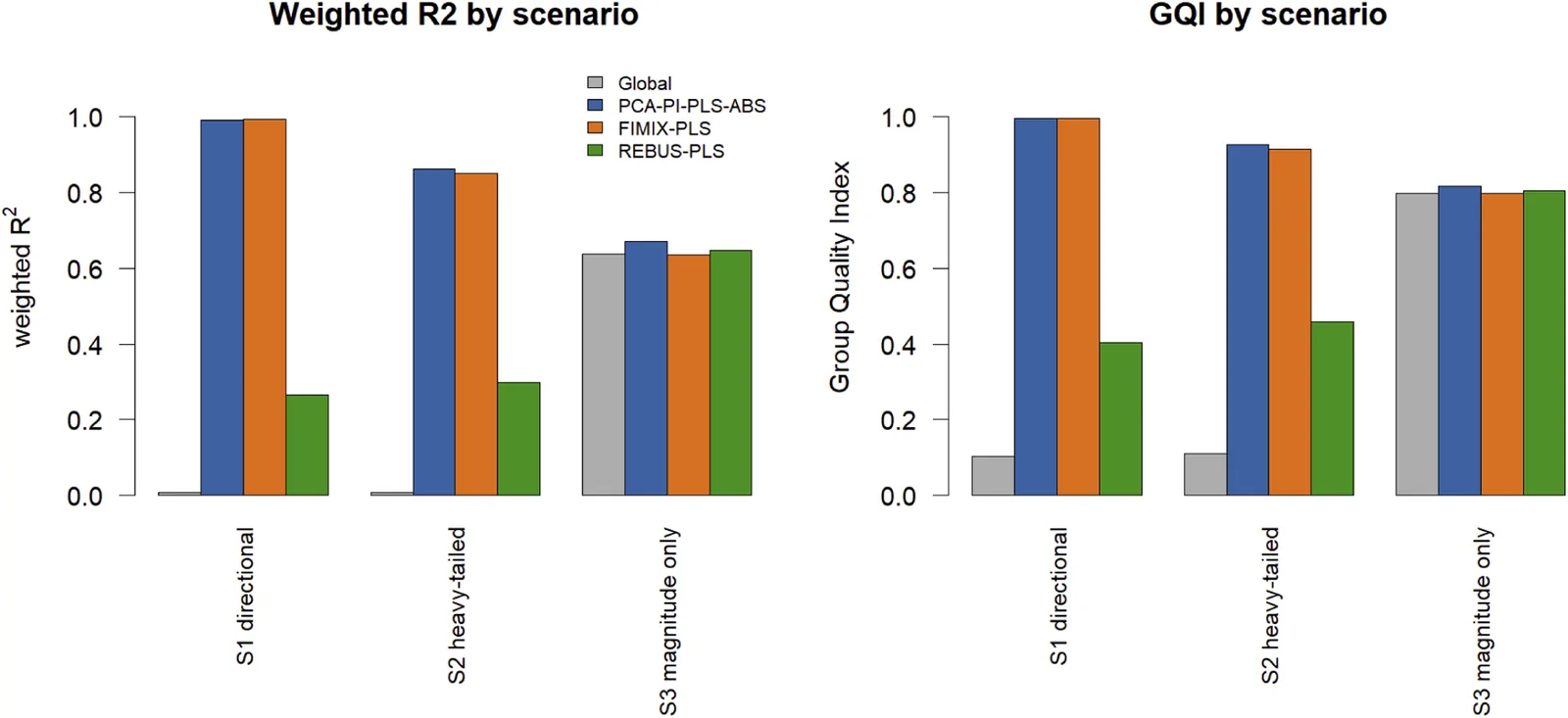
$R_{weighted}^{2}$ and group quality index of the three segmentation methods across the three simulation scenarios.

### Behaviour in more complex structural models

Three designs were used to examine whether the angular criterion remains informative when the structural model is more complex. D1 has two exogenous constructs, $\eta =\gamma_{1}\xi_{1}+\gamma_{2}\xi_{2}+\zeta$, with the first path reversing between groups ($\gamma_{1}=+1$ and −1) and the second homogeneous ($\gamma_{2}=+0.5$). D2 is a mediation chain $\xi \to \eta_{1}\to \eta_{2}$ in which only the second path reverses. D3 raises the number of reflective indicators per construct from three to ten, at n=400 so that the admissibility rule of FIMIX-PLS, which requires a smallest segment of at least ten times the number of indicators, can be met by a balanced two-segment solution. In D1 and D2 the proposed method and FIMIX-PLS are applied to the focal path after the remaining predictors have been partialled out as above, while REBUS-PLS is applied to the indicator blocks as they stand, since that procedure has no partial-residual generalisation.

Table 13 and Fig. 7 repeats the four-model comparison of the single-path benchmark in these three designs, using the same columns. The pattern is unchanged. PCA-PI-PLS-ABS and FIMIX-PLS both retain K=2 in every replication and are again almost indistinguishable in accuracy, 0.9452 against 0.9512 in D1, 0.9508 against 0.9552 in D2 and 0.9471 against 0.9497 in D3, with weighted coefficients of determination rising from an aggregate value near 0.01 to about 0.98 in all three designs. The gap that matters is again computational: the proposed method needs at most 0.002 s against 0.86 to 1.79 s for FIMIX-PLS, a factor of several hundred, and it requires no random restarts. REBUS-PLS remains the weakest and the least stable, its modal K being retained in only 37 to 57% of replications and its accuracy staying between 0.57 and 0.68, with gains of 0.27 to 0.45 against 0.97 or more for the other two. The angular criterion therefore transfers to branched and mediated models without loss, provided it is applied to the partial residuals of the focal path.

**Table 13:** Simulation benchmark of the three segmentation methods (50 replications per scenario, n = 200).

Design	Model	Modus K detected (Percentage)	Mean				
			Accuracy	$R_{weighted}^{2}$	GQI	Time (s)	R² gain
D1. Two exogenous constructs: $\gamma_{1}$ = (+1, −1), $\gamma_{2}$ = +0.5	Global (no segmentation)	1 (100%)	0.5	0.0081	0.1008	0	0
D1. Two exogenous constructs: $\gamma_{1}$ = (+1, −1), $\gamma_{2}$ = +0.5	PCA-PI-PLS-ABS (proposed)	2 (100%)	0.9452	0.9832	0.886	7e-04	0.9751
D1. Two exogenous constructs: $\gamma_{1}$ = (+1, −1), $\gamma_{2}$ = +0.5	FIMIX-PLS	2 (100%)	0.9512	0.9838	0.8867	0.8847	0.9758
D1. Two exogenous constructs: $\gamma_{1}$ = (+1, −1), $\gamma_{2}$ = +0.5	REBUS-PLS	2 (37%)	0.5708	0.2765	0.4141	0.0073	0.2684
D2. Mediation chain ξ → $\eta_{1}$ → $\eta_{2}$: $\beta_{2}$ = (+1, −1)	Global (no segmentation)	1 (100%)	0.5	0.0108	0.1168	0	0
D2. Mediation chain ξ → $\eta_{1}$ → $\eta_{2}$: $\beta_{2}$ = (+1, −1)	PCA-PI-PLS-ABS (proposed)	2 (100%)	0.9508	0.9872	0.9909	0	0.9763
D2. Mediation chain ξ → $\eta_{1}$ → $\eta_{2}$: $\beta_{2}$ = (+1, −1)	FIMIX-PLS	2 (100%)	0.9552	0.9878	0.9912	0.9687	0.977
D2. Mediation chain ξ → $\eta_{1}$ → $\eta_{2}$: $\beta_{2}$ = (+1, −1)	REBUS-PLS	2 (37%)	0.6383	0.4208	0.5405	0.006	0.4099
D3. Larger measurement blocks: 10 indicators per construct, n=400	Global (no segmentation)	1 (100%)	0.5	0.0044	0.0766	0	0
D3. Larger measurement blocks: 10 indicators per construct, n=400	PCA-PI-PLS-ABS (proposed)	2 (100%)	0.9471	0.9823	0.9614	0.0017	0.9779
D3. Larger measurement blocks: 10 indicators per construct, n=400	FIMIX-PLS	2 (100%)	0.9497	0.983	0.9618	1.7907	0.9786
D3. Larger measurement blocks: 10 indicators per construct, n=400	REBUS-PLS	2 (37%)	0.6632	0.4067	0.486	0.0163	0.4023

**Fig. 7:**
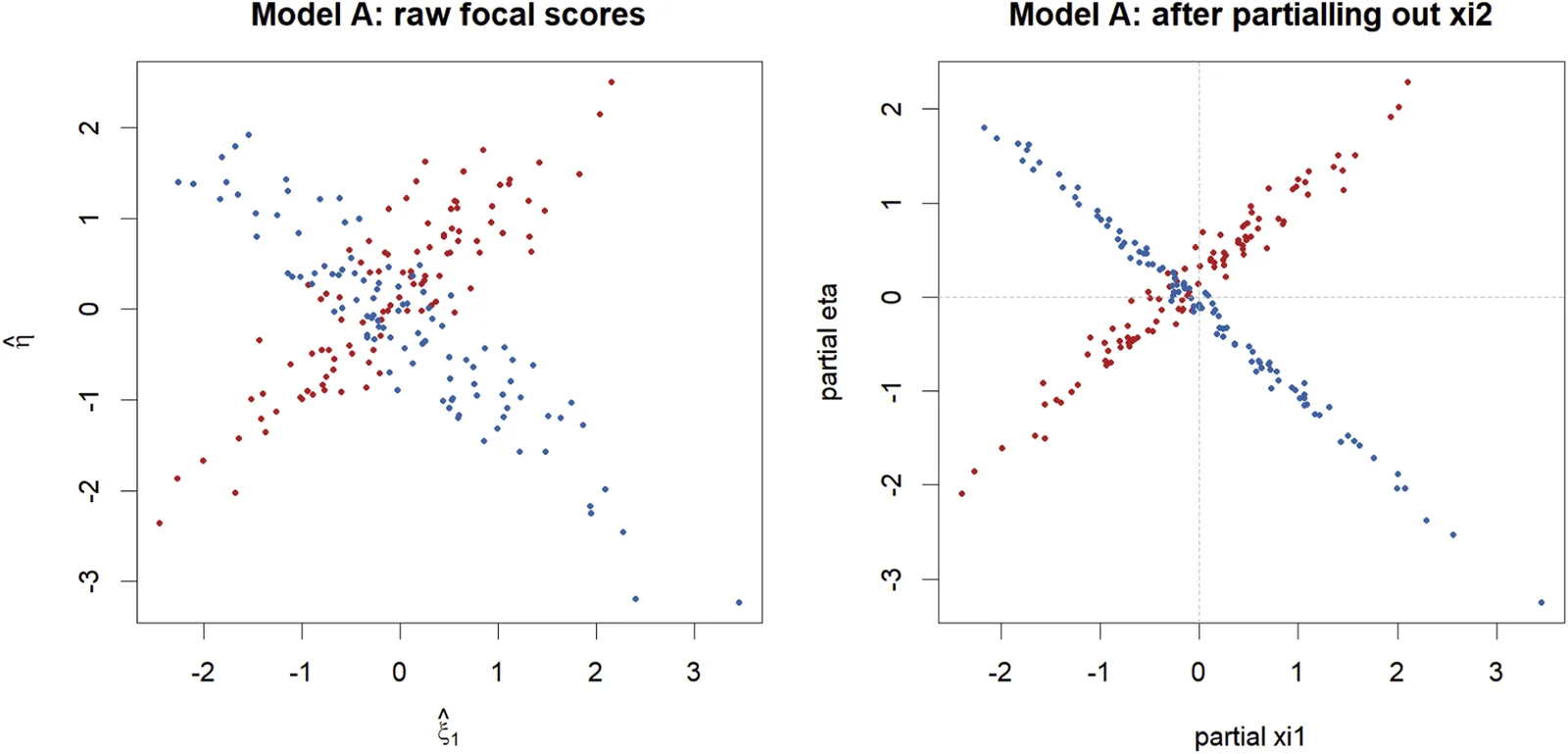
Focal latent scores before (left) and after (right) partialling out the second exogenous construct.

Table 14 applies the same acceptance protocol used for the single-path case and for the real data, so that the complex designs are held to an identical standard. The homogeneous residual bootstrap on $R_{gain}^{2}$ rejects in every replication of all three designs, with a median p−value of 0.0099, so the recovered structure is not an artefact of partitioning a homogeneous cloud. The weighted coefficient of determination replicates essentially exactly out of sample, 0.9834 against 0.9832 in D1, 0.9876 against 0.9872 in D2 and 0.9829 against 0.9823 in D3, which rules out a selection effect from estimating the local models inside the clusters used to define them. The half-sample Jaccard indices lie between 0.979and 0.984 for both segments in all three designs, comfortably above the 0.75 threshold. All three designs therefore satisfy both acceptance criteria simultaneously.

**Table 14:** Simulation Benchmark of the three segmentation methods (50 replications per scenario, n = 200).

Design	HBS median p	HBS rejection rate	$R_{weighted}^{2}$ in-sample	$R_{weighted}^{2}$ out-of-sample	Jaccard segment 1	Jaccard segment 2
D1. Two exogenous constructs: gamma1 = (+1, −1), gamma2 = +0.5	0.0099	1	0.9832	0.9834	0.979	0.98
D2. Mediation chain xi -> eta1 -> eta2: beta2 = (+1, −1)	0.0099	1	0.9872	0.9876	0.984	0.984
D3. Larger measurement blocks: 10 indicators per construct, n = 400	0.0099	1	0.9823	0.9829	0.983	0.983

### The angular definition put to the test

The argument above is analytic, so it was also verified numerically under the same evaluation columns used elsewhere in the paper. Four designs were run with 50 replications each at n=500. Because the angular separation is controlled by reliability rather than by the size of the coefficients, the designs vary the two things that actually move it. D1 is a balanced sign reversal with highly reliable indicators, which realises the largest separation the method can ever face, $\Delta \theta =89.76^{\circ }$, with only 2.1% of observations near the $\pm 90^{\circ }$ endpoints. D2 lowers the loadings to λ=0.6 and raises both disturbances to 0.6, which narrows the separation to $78.33^{\circ }$ and multiplies fivefold the share near the endpoints, to 10.3%. D3 returns to reliable indicators but makes the subgroups unequal, 400 against 100. D4 lowers reliability further, λ=0.5 with disturbances of 0.8, bringing the separation down to $66.73^{\circ }$ and 12.9% of observations near the endpoints, which is where detectability begins to bind. Within a design the two rows differ in one respect only, the definition of θ; standardization, euclidean distance, ward linkage and the farthest-distance rule for K are held fixed, so any difference is attributable to the angular definition alone.

In every design the two definitions differ by an order of magnitude in the substantive columns while costing the same computing time, about 0.004 s, so nothing whatever is bought by the change. At the largest separation available, D1, the accuracy is 0.9637 for arctan against 0.6040 for atan2, the $R_{weighted}^{2}$ is 0.9918 against 0.1817 and the gain over the aggregate model is 0.9883 against 0.1782 (See Table 15 and Fig. 8). As reliability falls the proposed method degrades gracefully while the alternative does not move at all: the accuracies are 0.7952 and 0.5576 in D2, and 0.7162 and 0.5515 in D4 where the separation is down to $66.73^{\circ }$, so atan2 never rises more than 0.11 above its own chance level anywhere on the ladder while arctan still recovers three-quarters of the true classification at the point where detectability begins to bind. D3 is the sharpest case. There atan2 scores 0.8000, and because the groups are split 400 against 100 that figure is exactly the share of the majority group, which is the chance level of accuracy for this design; the segmentation therefore carries no information whatever, and its gain of −0.1300 is NEGATIVE, meaning that the partition explains less of the endogenous construct than the aggregate model it replaces. What separates the two definitions is not the selection rule, since arctan retains K=2 in every replication of every design and atan2 in 86 to 100%, but what the segments contain, and Fig. 8 shows this directly. The left and centre columns are almost indistinguishable in all four rows; the right column is not. Because $\beta_{A}=+1$ sends units with ${\overset{ˆ}{\xi }}_{i}>0$ to $+45^{\circ }$ and units with ${\overset{ˆ}{\xi }}_{i}<0$ to $-135^{\circ }$, a two-segment cut pairs one arm of each subgroup with one arm of the other, and the resulting partition coincides with the sign of ${\overset{ˆ}{\eta }}_{i}$ in 99%, 97%, 100% and 79% of units across the four rows: it is a horizontal split of the scatter rather than a separation of the two lines. Each recovered segment therefore holds an even mixture of the two true subgroups, 52% against 48% in D1, and the local coefficients collapse from +0.996 and −0.995 to +0.071 and +0.089, that is back to the aggregate value the segmentation was meant to decompose. In D3 both recovered segments carry the same 80 to 20 mixture as the population itself, which is precisely why the accuracy there settles at the chance value of 0.8000.

**Table 15:** Direct comparison of the two angular definitions under identical clustering steps (50 replications per design, n = 500).

Design	Angular definition	Modus K detected (Percentage)	Mean				
			Accuracy	$R_{weighted}^{2}$	GQI	Time (s)	R² gain
D1. Sign reversal, balanced: β=(+1,−1), n=(250,250)	$\theta =\arctan(\frac{\hat{\eta }}{\hat{\xi }+\epsilon })$ [retained]	2 (100%)	0.9637	0.9918	0.9941	0.0042	0.9883
D1. Sign reversal, balanced: β=(+1,−1), n=(250,250)	$\theta =\text{atan}2(\hat{\eta },\hat{\xi })$ [reviewer's proposal]	2 (86%)	0.604	0.1817	0.2938	0.0034	0.1782
D2. Low reliability, dispersed angles: λ=0.6, $\sigma_{\zeta }=\sigma_{\epsilon }=0.6$	$\theta =\arctan(\frac{\hat{\eta }}{\hat{\xi }+\epsilon })$ [retained]	2 (100%)	0.7952	0.7283	0.7671	0.0044	0.7252
D2. Low reliability, dispersed angles: λ=0.6, $\sigma_{\zeta }=\sigma_{\epsilon }=0.6$	$\theta =\text{atan}2(\hat{\eta },\hat{\xi })$ [reviewer's proposal]	2 (98%)	0.5576	0.0689	0.1842	0.0034	0.0659
D3. Sign reversal, unbalanced: β=(+1,−1), n=(400,100)	$\theta =\arctan(\frac{\hat{\eta }}{\hat{\xi }+\epsilon })$ [retained]	2 (100%)	0.9668	0.9919	0.9941	0.0034	0.6245
D3. Sign reversal, unbalanced: β=(+1,−1), n=(400,100)	$\theta =\text{atan}2(\hat{\eta },\hat{\xi })$ [reviewer's proposal]	2 (100%)	0.5	0.2374	0.4772	0.0048	−0.13
D4. Near the detectability boundary: λ=0.5, $\sigma_{\zeta }=\sigma_{\epsilon }=0.8$	$\theta =\arctan(\frac{\hat{\eta }}{\hat{\xi }+\epsilon })$ [retained]	2 (100%)	0.7162	0.5789	0.6229	0.0046	0.576
D4. Near the detectability boundary: λ=0.5, $\sigma_{\zeta }=\sigma_{\epsilon }=0.8$	$\theta =\text{atan}2(\hat{\eta },\hat{\xi })$ [reviewer's proposal]	2 (98%)	0.5515	0.0528	0.1591	0.0036	0.0498

**Fig. 8:**
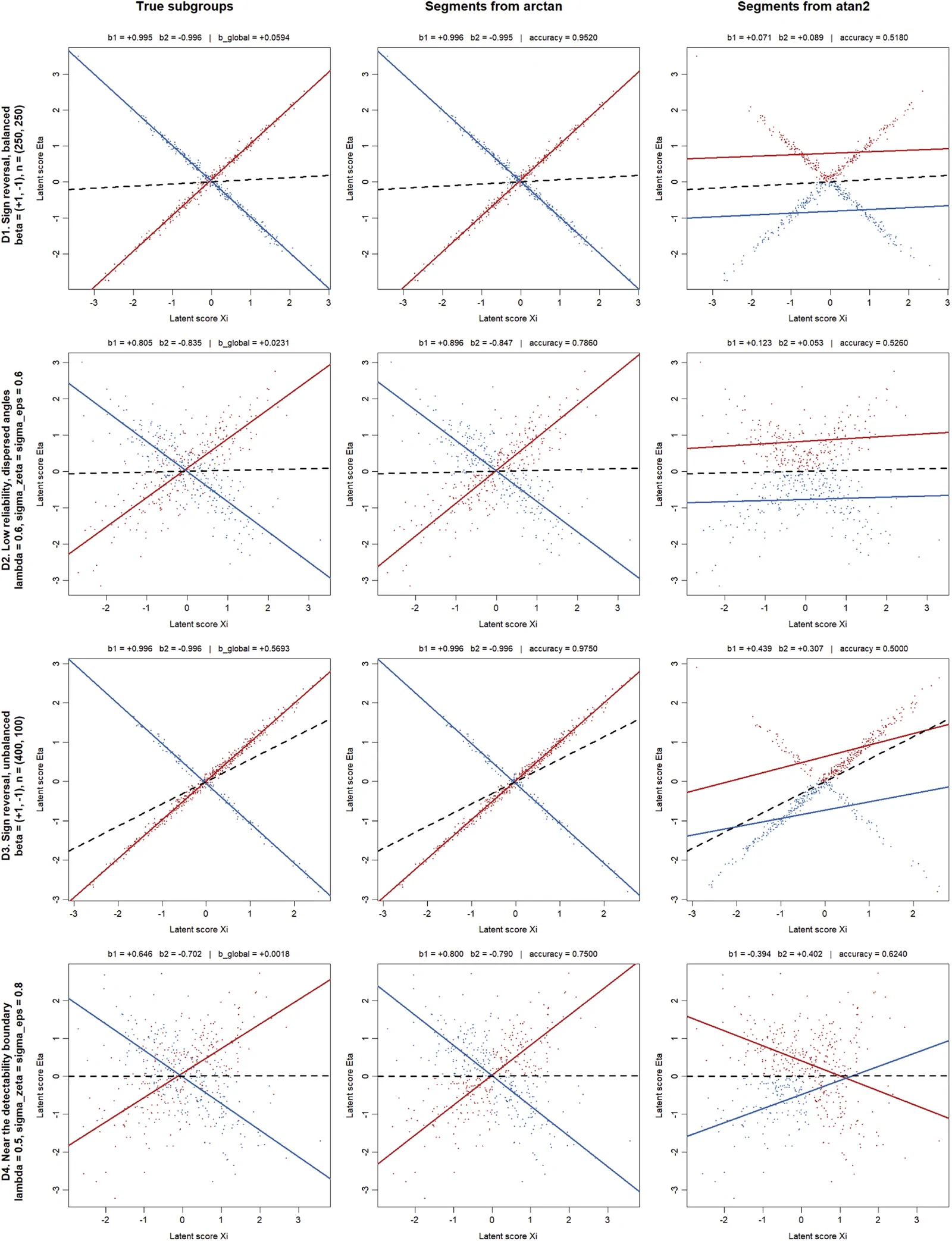
Latent scores of one replication of each design (n = 500): the true subgroups (left), the segments recovered from arctan (centre) and the segments recovered from atan2 (right). Solid lines are the local fits, the dashed line the aggregate fit; b1 and b2 are the local coefficients of the two segments.

Table 16 applies the acceptance protocol to both definitions with K fixed at two. Under arctan every design passes all four conditions: the median bootstrap p-value is 0.0099, the smallest value attainable with B=100 draws; the rejection rate at the nominal 5% level is 100%; the out-of-sample $R_{weighted}^{2}$ matches the in-sample value to within 0.005 in D1 to D3 and is 0.040 higher in D4, so there is no sign of over-fitting anywhere; and the Jaccard indices lie between 0.871 and 0.991. Under atan2 the protocol refuses the solution in three of the four designs, where the bootstrap does not reject at all, with median p-values of 0.8465, 0.8020 and 0.7574 and rejection rates of 0.25, 0.25 and 0.10, and where the out-of-sample $R_{weighted}^{2}$ falls to roughly half of an already small in-sample value: from 0.1121 to 0.0659, from 0.0668 to 0.0342 and from 0.0465 to 0.0304. The fourth design is the instructive one. In the unbalanced case the bootstrap rejects with p=0.0099 in every replication, yet the $R_{weighted}^{2}$is only 0.2459 against 0.9918 under arctan and lies below the aggregate $R^{2}$, so a partition that is formally significant against the homogeneous null still explains less than the model it replaces. That case is what makes the requirement $\Delta R^{2}>0$ necessary rather than decorative. The Jaccard indices make the same point from the opposite direction: under atan2 they range from 0.807 to 0.954, comfortably above the conventional threshold, so a wrong partition can be entirely reproducible. Stability is necessary but not sufficient, and only the conjunction of the four conditions distinguishes a segmentation that recovers a mechanism from one that merely repeats itself.

**Table 16:** Diagnostic acceptance protocol applied under both angular definitions (20 replications per cell, n = 500, K = 2).

Design	Angular definition	HBS median p	HBS rejection rate	$R_{weighted}^{2}$ in-sample	$R_{weighted}^{2}$ out-of-sample	Jaccard segment 1	Jaccard segment 2
D1. Sign reversal, balanced: β=(+1,−1), n=(250,250)	$\theta =\arctan(\frac{\hat{\eta }}{\hat{\xi }+\epsilon })$ [retained]	0.0099	1	0.9919	0.9921	0.988	0.988
D1. Sign reversal, balanced: β=(+1,−1), n=(250,250)	$\theta =\text{atan}2(\hat{\eta },\hat{\xi })$ [reviewer's proposal]	0.8465	0.25	0.1121	0.0659	0.9	0.907
D2. Low reliability, dispersed angles: λ=0.6, $\sigma_{\zeta }=\sigma_{\epsilon }=0.6$	$\theta =\arctan(\frac{\hat{\eta }}{\hat{\xi }+\epsilon })$ [retained]	0.0099	1	0.7415	0.7468	0.928	0.928
D2. Low reliability, dispersed angles: λ=0.6, $\sigma_{\zeta }=\sigma_{\epsilon }=0.6$	$\theta =\text{atan}2(\hat{\eta },\hat{\xi })$ [reviewer's proposal]	0.802	0.25	0.0668	0.0342	0.831	0.819
D3. Sign reversal, unbalanced: β=(+1,−1), n=(400,100)	$\theta =\arctan(\frac{\hat{\eta }}{\hat{\xi }+\epsilon })$ [retained]	0.0099	1	0.9918	0.9919	0.991	0.97
D3. Sign reversal, unbalanced: β=(+1,−1), n=(400,100)	$\theta =\text{atan}2(\hat{\eta },\hat{\xi })$ [reviewer's proposal]	0.0099	1	0.2459	0.2242	0.947	0.954
D4. Near the detectability boundary: λ=0.5, $\sigma_{\zeta }=\sigma_{\epsilon }=0.8$	$\theta =\arctan(\frac{\hat{\eta }}{\hat{\xi }+\epsilon })$ [retained]	0.0099	1	0.5697	0.6097	0.875	0.871
D4. Near the detectability boundary: λ=0.5, $\sigma_{\zeta }=\sigma_{\epsilon }=0.8$	$\theta =\text{atan}2(\hat{\eta },\hat{\xi })$ [reviewer's proposal]	0.7574	0.1	0.0465	0.0304	0.827	0.807

### Robustness under less favourable conditions

The principal simulation uses highly reliable indicators and small disturbances, so the extent to which the reported performance depends on that configuration must be established. Eleven conditions were run with 100 replications each, holding β=(+1,−1) throughout and varying ONE design feature at a time except in the final combined case: the loadings λ, loadings that differ within a block and between the two groups, the measurement error $\sigma_{\varepsilon }$, the structural disturbance $\sigma_{\zeta }$, the balance of the two groups and the total sample size. Varying one feature at a time is what separates this experiment from the reliability ladder of the previous sub-section, which moved λ, $\sigma_{\varepsilon }$ and $\sigma_{\zeta }$ together in order to keep the comparison between the two angular definitions clean; here the object is to identify which feature actually binds. The columns are those used throughout the paper, so this table can be read against the benchmark and the real-case application directly.

Table 17 shows that the procedure degrades gradually and predictably rather than breaking down, and that the degradation follows the angular mechanism set out in the method section. The farthest-distance rule retains K=2 in 100% of replications in every one of the eleven conditions, so nothing that follows is an artefact of a mis-selected number of segments. Reflective loadings matter far less than might be feared: lowering λ uniformly from 0.90 to 0.45 reduces the accuracy only from 0.9530 to 0.9414, letting the loadings differ within a block (0.85/0.70/0.55) gives 0.9493, and letting them differ between the two groups (0.90 against 0.60) gives 0.9496. The binding constraints are noise: raising $\sigma_{\varepsilon }$ to 0.50 gives 0.8920, and raising $\sigma_{\zeta }$ to 0.50 and 1.00 gives 0.8822 and 0.7999. This is exactly what the standardised geometry predicts, because noise is what compresses the angle: the true angular separation falls from $89.76^{\circ }$ at the baseline to $87.24^{\circ }$, $86.79^{\circ }$ and $79.07^{\circ }$ across those three conditions, while the share of observations lying beyond $|\theta |>75^{\circ }$ rises from 2.3% to 5.5%, 6.0% and 10.2%. Sample size matters far less than might be expected, the accuracy moving only from 0.9360 at n=50 to 0.9668 at n=1,000, which is consistent with the geometric argument developed in the sub-section on the operating region: what the method needs is angular separation rather than observations. Two conditions separate accuracy from $\Delta R^{2}$ and are worth reading carefully. With unequal groups at a ratio of 180:20 the accuracy is unchanged at 0.9638, but $\Delta R^{2}$ falls to 0.3622 simply because the minority segment carries little weight in a size-weighted average; and when the loadings differ between the groups the gain falls to 0.846 because the weaker block leaves the aggregate model with an $R^{2}$ of 0.143 rather than nearly zero, so there is less to recover. Even in the combined worst case, with λ=0.60, $\sigma_{\zeta }=0.50$, $\sigma_{\varepsilon }=0.50$ and a 150:50 split, the accuracy is 0.8214 with a gain of 0.5909 and a GQI of 0.813. Computing time never exceeds 0.0147 s, which is the cost at n=1,000. Fig. 9 shows the same four points of that ladder as scatter plots: the 2 p.m. of the cross blur as the noise rises, and in the worst case the aggregate line is no longer flat, its coefficient being +0.402, yet the sign reversal is still recovered with local coefficients of +0.877 and −0.868.

**Table 17:** Robustness of PCA-PI-PLS-ABS under less favourable measurement and design conditions (100 replications per condition, β = (+1, −1) throughout).

Condition	K detected	Accuracy	$R_{weighted}^{2}$	GQI	Time (s)	R² gain
C1. Baseline: λ=0.90, $\sigma_{\zeta }=0.10$, $\sigma_{\varepsilon }=0.10$, n=(100,100)	2 (100%)	0.953	0.9911	0.9938	0.0017	0.9815
C2. Weak loadings: λ=0.45	2 (100%)	0.9414	0.9809	0.9834	0.0016	0.9713
C3. Heterogeneous loadings within block: 0.85/0.70/0.55	2 (100%)	0.9493	0.9883	0.9909	0.002	0.9786
C4. Heterogeneous loadings across groups: 0.90 vs 0.60	2 (100%)	0.9496	0.9892	0.9918	0.0013	0.8463
C5. Large measurement error: $\sigma_{\varepsilon }=0.50$	2 (100%)	0.892	0.9172	0.9188	0.0018	0.9079
C6. Large structural noise: $\sigma_{\zeta }=0.50$	2 (100%)	0.8822	0.9038	0.9495	0.0019	0.8946
C7. Very large structural noise: $\sigma_{\zeta }=1$	2 (100%)	0.7999	0.7402	0.86	0.0022	0.7319
C8. Unequal groups:n=(180,20)	2 (100%)	0.9638	0.9911	0.9938	0.0013	0.3622
C9. Small sample: n=(25,25)	2 (100%)	0.936	0.9871	0.992	9e-04	0.9391
C10. Large sample: n=(500,500)	2 (100%)	0.9668	0.992	0.9942	0.0163	0.9903
C11. Combined worst case: λ=0.60, $\sigma_{\zeta }=0.50$, $\sigma_{\varepsilon }=0.50$, n=(150,50)	2 (100%)	0.8214	0.7781	0.813	0.0018	0.5909

**Fig. 9:**
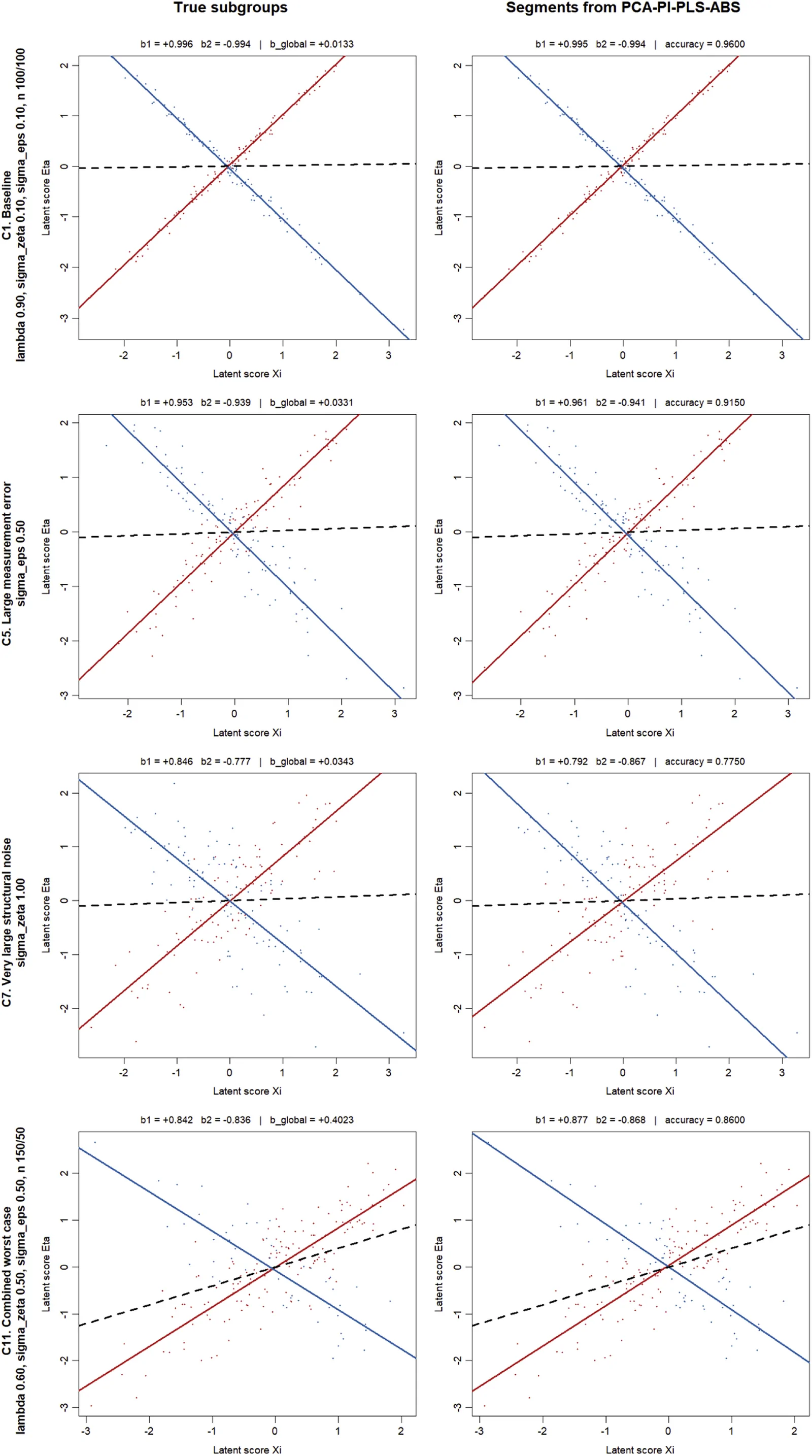
Latent scores under four of the eleven conditions, forming a degradation ladder: the true subgroups (left) against the segments recovered by PCA-PI-PLS-ABS (right). Solid lines are the local fits, the dashed line the aggregate fit.

Table 18 applies the acceptance protocol of the method section to each condition, with K fixed at two. The result is uniform: the median bootstrap p-value is 0.0099, the smallest value attainable with B=100 draws, and the rejection rate at the nominal 5% level is 1.00 in all eleven conditions, so the test retains full power against the homogeneous null even where the classification itself has become imprecise. The out-of-sample $R_{weighted}^{2}$ tracks the in-sample value throughout, the largest discrepancy being 0.015 and its direction usually upward, which rules out over-fitting as an explanation of the gains reported in Table 17; the only condition in which the out-of-sample value is lower is the smallest sample, n=50, and even there the drop is 0.005. Both Jaccard indices exceed the conventional threshold of 0.75 in every condition, ranging from 0.910 to 0.995, and the two lowest values belong to the minority segments of the two unbalanced designs, which is where a stability index should be weakest. Taken with Table 18 this settles the reviewer's concern in the direction the reviewer suspected but with a narrower conclusion than feared: measurement quality does govern performance, but through the angular separation alone, and the acceptance protocol continues to certify the solution across the entire range examined (Fig. 10).

**Table 18:** Acceptance protocol applied to PCA-PI-PLS-ABS under each robustness condition (20 replications per condition, K = 2).

Condition	HBS median p	HBS rejection rate	$R_{weighted}^{2}$ in-sample	$R_{weighted}^{2}$out-of-sample	Jaccard segment 1	Jaccard segment 2
C1. Baseline: λ=0.90, $\sigma_{\zeta }=0.10$, $\sigma_{\varepsilon }=0.10$, n 100/100	0.0099	100%	0.9913	0.9914	0.988	0.988
C2. Weak loadings: λ=0.45	0.0099	100%	0.9807	0.9807	0.986	0.986
C3. Heterogeneous loadings within block: 0.85/0.70/0.55	0.0099	100%	0.9883	0.9884	0.985	0.985
C4. Heterogeneous loadings across groups: 0.90 vs 0.60	0.0099	100%	0.9889	0.9892	0.992	0.991
C5. Large measurement error: $\sigma_{\varepsilon }=0.50$	0.0099	100%	0.9104	0.9153	0.959	0.96
C6. Large structural noise: $\sigma_{\zeta }=0.50$	0.0099	100%	0.8991	0.9051	0.944	0.944
C7. Very large structural noise: $\sigma_{\zeta }=1.00$	0.0099	100%	0.737	0.7517	0.932	0.928
C8. Unequal groups: n 180/20	0.0099	100%	0.9909	0.991	0.995	0.966
C9. Small sample: n 25/25	0.0099	100%	0.9861	0.9811	0.989	0.989
C10. Large sample: n 500/500	0.0099	100%	0.9921	0.9923	0.985	0.985
C11. Combined worst case: λ=0.60, $\sigma_{\zeta }=0.50$, $\sigma_{\varepsilon }=0.50$, n 150/50	0.0099	100%	0.7788	0.7859	0.954	0.91

**Fig. 10:**
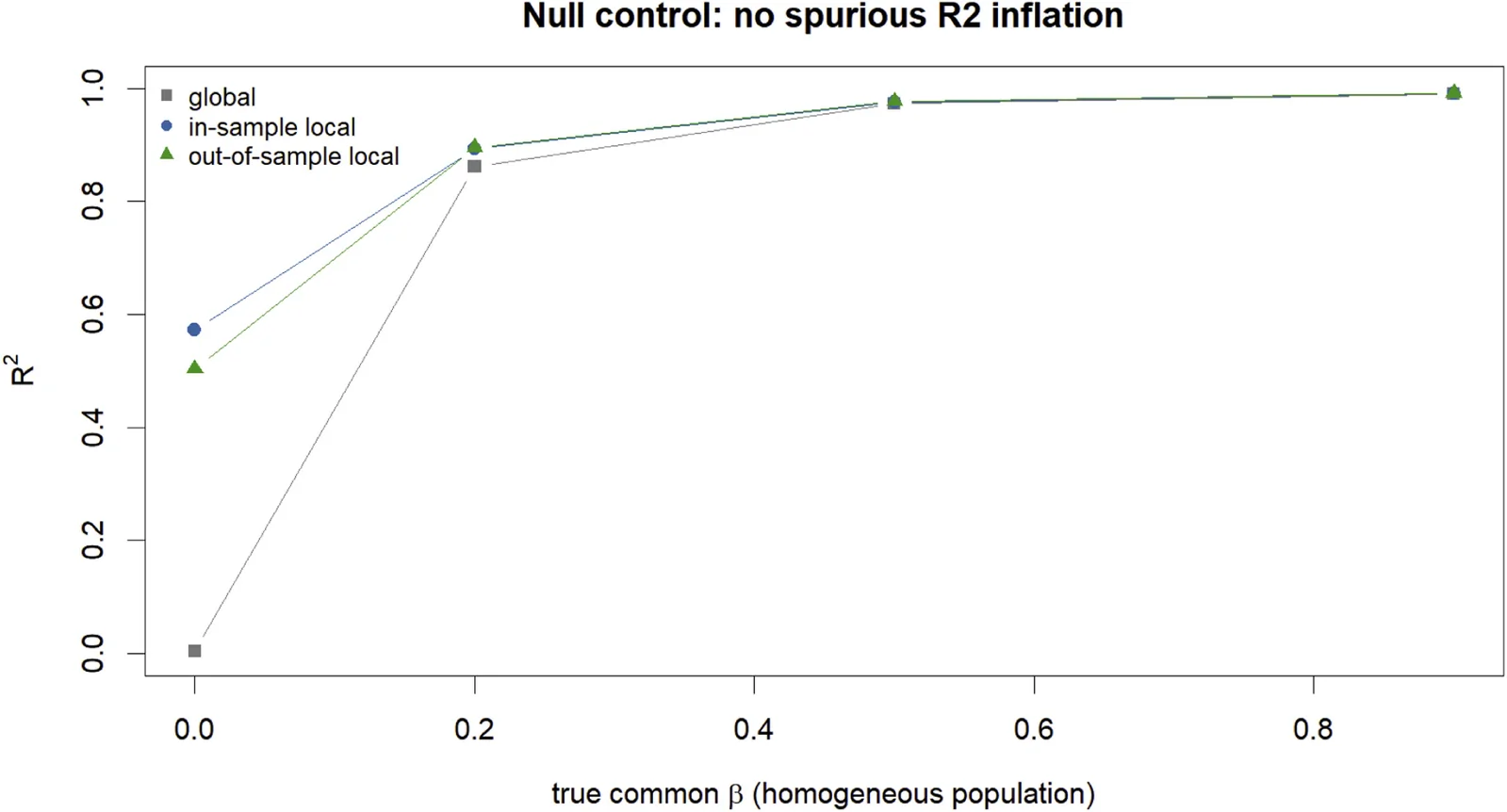
Local R² in sample and out of sample under a homogeneous population, as a function of the true common β.

### Out-of-sample validation and a test for genuine heterogeneity

Because the segments are constructed from a function of the latent scores and the local models are then estimated on those same scores, the increase in $R_{weighted}^{2}$ could in principle be a selection effect. Two safeguards are therefore built into the procedure. The first is out-of-sample estimation: the clusters are formed on a random half of the observations, the remaining observations are assigned by a nearest-centroid rule in the angular space, and $R_{weighted}^{2}$ is computed on the held-out half alone. The second is a formal test, whose null hypothesis must be a single homogeneous population with the same path coefficient as the aggregate model rather than the absence of any relationship. We realise it as a residual bootstrap, generating $\eta^{*}={\overset{ˆ}{\beta }}_{\text{global}}\overset{ˆ}{\xi }+\varepsilon^{*}$ with $\varepsilon^{*}$ resampled from the residuals of the aggregate model and repeating the segmentation on each replicate, with $\Delta R^{2}$ as the test statistic. Six conditions were examined with 50 replications each.

Table 19 establishes three things. First, the gain is not an artefact of estimating a model inside the clusters used to define it: at β=(+1,−1) the $R_{weighted}^{2}$ is 0.9910 in sample and 0.9911 out of sample, and at β=(+1,−0.25) it is 0.9463 and 0.9489. Second, the test behaves correctly. Its power is 1.00 in both directional conditions, while under a genuinely homogeneous population at β=(+0.9,+0.9) the rejection rate is 4%, matching the nominal 5% level, and at β=(0,0) it is 0.02 even though the in-sample $R_{weighted}^{2}$ there rises to 0.3623 from an aggregate $R^{2}$ of 0.0057. That last row is the important one: partitioning an unstructured cloud does inflate the local fit, out of sample as well as in sample, which is precisely why the test is a required step of the method rather than an optional check. Third, the test is conservative and has one blind spot that should be reported openly: when one subgroup has no relationship at all, at β=(+1,0), the rejection rate is zero, because such a mixture is nearly observationally equivalent to a single population with larger dispersion. A non-significant result should accordingly be read as an absence of evidence rather than as evidence of homogeneity.

**Table 19:** Out-of-sample validation and the test for genuine heterogeneity (50 replications per condition).

Condition	R² global	$R_{weighted}^{2}$ in-sample	$R_{weighted}^{2}$ out-of-sample	Median test p	Rejection rate at 5%
Directional heterogeneity, β=(+1,−1)	0.0072	0.991	0.9911	0.0099	100%
Directional heterogeneity, β=(+1,−0.25)	0.2549	0.9463	0.9489	0.0099	100%
One subgroup with no relationship, β=(+1,0)	0.488	0.5092	0.5286	0.8614	0%
Magnitude-only heterogeneity, β=(+1,+0.5)	0.8899	0.8941	0.8867	0.2673	22%
Homogeneous population, β=(+0.9,+0.9)	0.9903	0.9637	0.9628	0.6238	4%
Homogeneous population with no relationship, β=(0,0)	0.0057	0.3623	0.4084	0.5	2%

### Stability of the clustering solution and the uncertainty of $\overset{ˆ}{K}$

Hierarchical clustering always returns a partition, so the stability of the solution and the uncertainty attaching to the retained number of segments must be examined separately from its accuracy. Three quantities were computed over 50 replications in each of seven conditions: the half-sample Jaccard index, obtained by re-clustering random half-samples and matching each segment to its best counterpart; the proportion of replications in which the farthest-distance rule retains the true K; and the p-value of the homogeneity test described above.

Table 20 supports the two acceptance criteria adopted here and identifies one limitation. In the five conditions with genuine directional heterogeneity the Jaccard index lies between 0.932 and 0.984, the correct K is retained in every replication, and the test is significant. In the collinear condition β=(+1,+0.5), which lies outside the operating region, all three indicators fail together: the accuracy is 0.5951, the Jaccard index falls to 0.734, the correct K is retained in only 44% of replications and the test does not reject. The choice of agglomeration rule is not critical, since Ward.D2, Ward.D, average, complete and centroid linkage all give accuracies between 0.9526 and 0.9551 and all retain K=2 in every replication, whereas single linkage is clearly inferior at 0.8462 and 0.56; Ward.D2 is retained as the default. The limitation concerns K>2: with three latent classes at β=(+1,0,−1) the transformation still separates the classes well, with a accuracy of 0.8977 when the tree is cut at K=3, but the farthest-distance rule selects the correct *K* in only 8% of replications, because on the linear θ scale the class with β=0 lies between the other two and the first agglomeration gap therefore dominates the second. For K>2 the gap rule must not be used alone, K should be chosen by scanning $K=2,\ldots ,K_{\text{max}}$ with the test and the stability index. Above all, stability alone is never sufficient evidence, since a stable partition also arises from a homogeneous cloud, and a partition is accepted here only when the test is significant and the Jaccard index is at least 0.75. As shown in Fig. 11, the use of single linkage yields accuracy that is still lower than the other methods; therefore, single linkage is not recommended. Ward.D2 achieves the highest segmentation accuracy, at 0.954.

**Table 20:** Stability of the clustering solution and recovery of the number of segments (50 replications per condition).

Condition	True K	Accuracy	Jaccard (min)	P(correct K)	Median test p
Baseline, beta = (+1, −1)	2	0.9535	0.984	100%	0.0164
Large structural noise, $\sigma_{\zeta }=0.50$	2	0.8841	0.955	100%	0.0164
Weak measurement, λ=0.60 and $\sigma_{\varepsilon }=0.50$	2	0.8472	0.932	100%	0.0164
Small sample, n 30/30	2	0.927	0.976	100%	0.0164
Unequal groups, n 180/20	2	0.9635	0.96	100%	0.0164
Collinear subgroups, β=(+1,+0.5)	2	0.5951	0.734	44%	0.2705
Three latent classes, β=(+1,0,−1)	3	0.8977	0.921	8%	0.0164

**Fig. 11:**
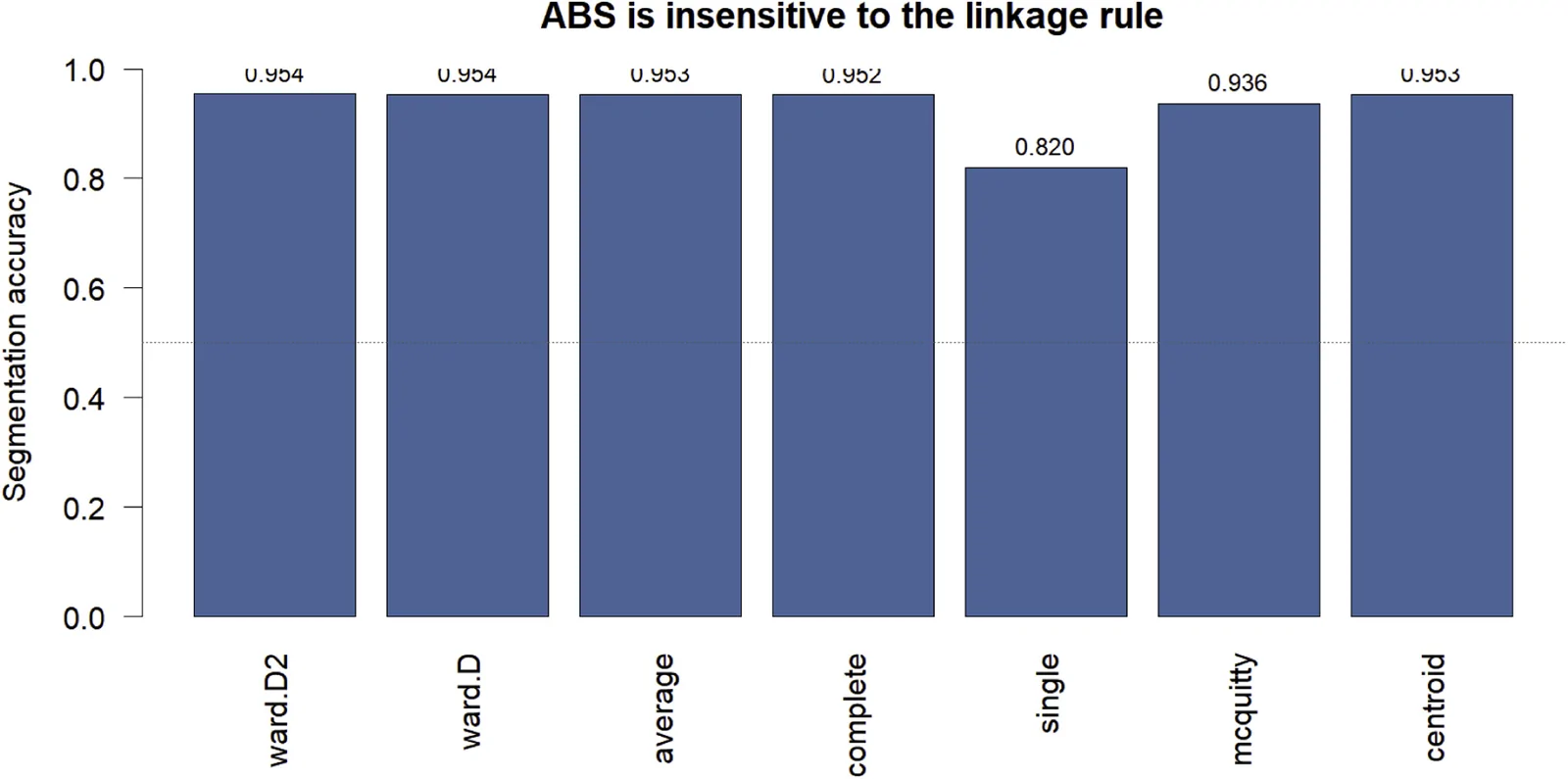
Segmentation accuracy under seven agglomeration rules.

### Operating region and the minimum detectable angular separation

The accuracy of the segmentation depends on how different the directions of the subgroup relationships are, and that dependence can be characterized explicitly. For two structural lines with coefficients $\beta_{A}$ and $\beta_{B}$ the true angular separation is,

$$\Delta \theta =\min\left(\left|\arctan\beta_{A}-\arctan\beta_{B}\right|,\;180^{\circ }-\left|\arctan\beta_{A}-\arctan\beta_{B}\right|\right)$$

the minimum reflecting the axial nature of the angles. Accuracy here is the plain proportion of units assigned to the correct subgroup after the best matching of segment labels to true groups. It was mapped as a function of Δθ over twelve values of $\beta_{B}$ and twelve design conditions with 60 replications per point, and the minimum detectable angular separation $\Delta \theta_{\text{min}}$ was defined as the smallest separation at which the mean accuracy reaches 0.80. One property of that measure must be stated before the results are read. The chance level of plain accuracy is not one-half but the share of the largest group, so it rises with imbalance: it is 0.50 for equal groups, 0.75 at a 150:50 split and 0.90 at 180:20. In the last of these the 0.80 threshold lies below chance, because a solution that simply follows the majority group already clears it; that condition was therefore excluded from the calibration and is marked as not interpretable in Table 21. Its nominal value, $\Delta \theta_{\text{min}}=1.47^{\circ }$, measures group imbalance and not detectability.

**Table 21:** Operating region: minimum detectable angular separation and the largest separable second-group coefficient.

n	λ	$\sigma_{\zeta }$	$\sigma_{\varepsilon }$	$\Delta \theta_{\text{min}}$ (deg)	$\beta_{B}$ max	Verdict
100/100	0.9	0.1	0.1	26.69	0.331	moderate
100/100	0.9	0.3	0.1	38.45	0.115	moderate
100/100	0.9	0.5	0.1	51.91	−0.121	narrow - opposite signs only
100/100	0.6	0.1	0.3	42.09	0.051	moderate
100/100	0.6	0.3	0.5	68.37	−0.432	narrow - opposite signs only
25/25	0.9	0.1	0.1	30.24	0.263	moderate
50/50	0.9	0.1	0.1	27.07	0.324	moderate
250/250	0.9	0.1	0.1	26.5	0.335	moderate
500/500	0.9	0.1	0.1	26.07	0.343	moderate
150/50	0.9	0.1	0.1	21.34	0.438	wide operating region
180/20	0.9	0.1	0.1	1.47	0.95	not interpretable - threshold below chance
250/250	0.6	0.5	0.5	76.6	−0.615	narrow - opposite signs only

Across the eleven remaining conditions $\Delta \theta_{\text{min}}$ is described accurately by,

$$\Delta \theta_{\text{min}}\approx 0.25848\;\sigma_{\theta }^{1.475}\;n_{\text{min}}^{0.071}\;\pi_{\text{min}}^{0.221}\;\left(\text{degrees}\right)$$

where $\sigma_{\theta }$ is the within-segment standard deviation of θ in degrees, $n_{\text{min}}$ the size of the smaller segment and $\pi_{\text{min}}$ its proportion. The fit has an adjusted $R^{2}$ of 0.9767 and a mean absolute error of $1.63^{\circ }$, and it validates on three conditions held out from the calibration design with errors of $1.5^{\circ }$, $0.7^{\circ }$ and $0.4^{\circ }$. The dominant term is the within-segment angular dispersion, with an elasticity of about 1.5, whereas sample size enters with an exponent of only 0.071; a naive $1/\sqrt{n}$ scaling is explicitly rejected because $\sigma_{\theta }$ already absorb the effect of n.

The exponent on $\pi_{\text{min}}$ requires comment, because its sign is not the one a reader would expect. It is positive, +0.221 with a p-value of 0.079, so the expression predicts a smaller required separation as the minority segment shrinks. This is a property of the accuracy measure rather than of the method: since the chance level rises with imbalance, an unbalanced design crosses the 0.80 threshold at a smaller true separation. The effect is visible directly in Table 21, where the 150:50 condition requires only $21.34^{\circ }$ against $26.69^{\circ }$ for the same design when balanced. Two consequences follow for applied use. The expression is accurate for approximately balanced designs and should be read as a lower bound when the minority segment is small; and because the 180:20 condition had to be excluded, the $\pi_{\text{min}}$ term is identified from a single unbalanced point and is not significant at the 5% level, so a user who needs a guarantee for a strongly unbalanced design should ignore that term and treat the balanced value as the requirement.

Table 21 converts the expression into an operating table by reporting, for each design condition, the largest second-group coefficient is still separable from $\beta_{A}=1.0$. At the baseline the requirement is $\Delta \theta_{\text{min}}=26.69^{\circ }$, which corresponds to $\beta_{B}\leq 0.331$; it rises to $38.45^{\circ }$ and $\beta_{B}\leq 0.115$ when $\sigma_{\zeta }=0.30$, to $51.91^{\circ }$ when $\sigma_{\zeta }=0.50$, at which point only opposite signs remain separable, and to $76.60^{\circ }$ under weak measurement combined with large disturbances (see also Fig. 12). Sample size matters little across the range examined, the requirement moving only from $30.24^{\circ }$ at n=50 to $26.07^{\circ }$ at n=1,000, which is the same conclusion the robustness table reached from the other direction. The expression is usable before an analysis is run, since $\sigma_{\theta }$ can be derived from an expected within-group correlation, and it accounts for the empirical result reported below, where only $70.3^{\circ }$ of separation is available against a required $79.33^{\circ }$. That survey is itself unbalanced, with a minority share of 0.213, so the positive $\pi_{\text{min}}$ exponent makes $79.33^{\circ }$ a lower bound; setting the $\pi_{\text{min}}$ term aside and reading the balanced requirement gives $95.79^{\circ }$, which exceeds the $90^{\circ }$ ceiling that the standardised geometry imposes on Δθ altogether. Either reading places the application outside the operating region for per-respondent classification, which is why the conclusions drawn from it are stated at segment level.

**Fig. 12:**
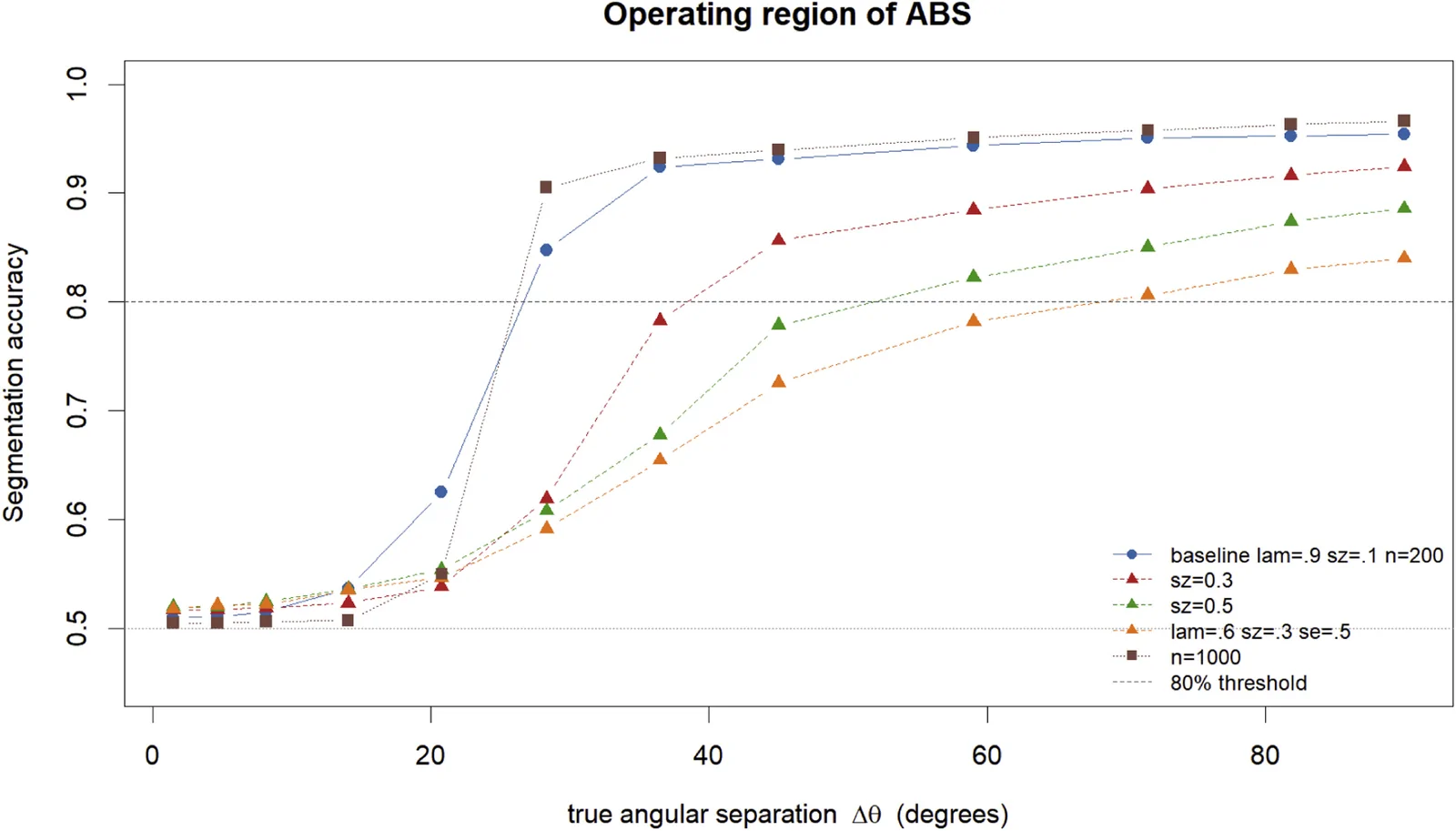
Accuracy as a function of the true angular separation Δθ under five design conditions.

### Computational environment

All analyses were carried out in R version 4.5.3 (2026-03-11 ucrt) on the x86_64-w64-mingw32 platform. The PCA-PI-PLS-ABS algorithm, including the Power Iteration routine, the angle-based transformation and the hierarchical clustering step, was implemented entirely with base R, using cov, cor, dist, hclust with Ward.D2 linkage, cutree and lm from the stats package; no third-party structural-equation-modelling package was required. The benchmark implementations of FIMIX-PLS and REBUS-PLS were likewise written in base R, so that the comparison is not affected by differences between packages. The empirical application reads the survey data from an Excel workbook using readxl version 1.5.0. Random number generation used the Mersenne-Twister generator with explicit seeds; the master seed is 20260904 and each Monte Carlo replication uses sequential seeds 1,…,R. The complete session information is provided as supplementary material.

### Practical guidance and diagnostic protocol

The validation supports a small set of default settings and a decision rule for accepting a segmentation, collected here so that the procedure can be applied without re-deriving them. The angular transformation uses the standardised θ of Eq. (7) on the linear scale with $\epsilon =10^{-6}$; the clustering uses Euclidean distance with Ward.D2 linkage; the 5% of observations with the smallest radius are trimmed beforehand; and a segment must contain at least 30 observations before a local model is estimated in it. Because the method depends on angular separation rather than on sample size, its feasibility can also be assessed in advance: an expected within-group correlation of 0.99, 0.90, 0.80 and 0.60 corresponds to $\sigma_{\theta }$ of $22.1^{\circ }$, $36.4^{\circ }$, $41.8^{\circ }$ and $47.0^{\circ }$, which substituted into the expression for $\Delta \theta_{\text{min}}$ gives the separation the design must provide. Since the largest possible separation is $90^{\circ }$, the procedure is infeasible once $\sigma_{\theta }$ exceeds roughly $45^{\circ }$, that is once the within-group $R^{2}$ falls below about 0.36.

Table 22 records each recommendation together with the analysis that supports it, and the decision rule that follows has eight steps. First, estimate the aggregate model; if its $R^{2}$ is high and its coefficient clearly non-zero, directional heterogeneity cannot be dominant. Second, compare the separation the design can be expected to provide with $\Delta \theta_{\text{min}}$, remembering that where the segments are expected to be unequal in size the expression understates the requirement, so the balanced value should be used instead. Third, run the segmentation with the settings above. Fourth, check that $R_{gain}^{2}>0$; a segmentation whose size-$R_{weighted}^{2}$ falls below the aggregate $R^{2}$ is discarded at once, whatever any test may say about it. Fifth, apply the homogeneous residual bootstrap to $R_{gain}^{2}$; if p≥0.05, do not report a segmentation, and note that neither the angular separation nor the sign reversal may be used as evidence in its place, since a homogeneous population produces both routinely. Sixth, compute the half-sample Jaccard index; a value below 0.75 is grounds for rejection, but a value above it is not by itself grounds for acceptance, since a partition can be perfectly reproducible and still wrong. Seventh, re-estimate the local coefficients and $R_{weighted,\text{out}}^{2}$ and report both, and require the out-of-sample value to stay close to the in-sample one. Eighth, only then interpret the segments substantively. An applied study should report the aggregate coefficient and $R^{2}$; $\sigma_{\theta }$, $\pi_{\text{min}}$ and the required $\Delta \theta_{\text{min}}$; the linkage rule, distance measure and rule used to select K; the size and local coefficient of every segment; the p−value of the homogeneity test; the Jaccard index of every segment; $R_{weighted,in}^{2}$ and $R_{weighted,\text{out}}^{2}$; the percentage of low-radius observations trimmed; and the R version, package versions and random seed.

**Table 22:** Recommended settings for applying PCA-PI-PLS-ABS and the analysis that supports each choice.

Parameter	Recommendation
Angular transformation	standardized $\theta =\arctan(\frac{\hat{\eta }}{\hat{\xi }+\epsilon })$, linear scale
Epsilon in arctan	$10^{-6}$; results invariant from $10^{-2}$ to $10^{-12}$
Distance measure	Euclidean on standardized θ
Linkage rule	Ward.D2 (or average / centroid)
Choice of K	largest dendrogram gap, then confirm by stability
Low-radius observations	trim the lowest 5% of radius r
Minimum cluster size	at least 30 observations, or 10% of n
Acceptance protocol (all four required)	$R_{gain}^{2}>0$; bootstrap p<0.05; $R_{weighted,\text{out}}^{2}$ close to $R_{weighted,in}^{2}$; Jaccard≥0.75
Evidence that the split is real	homogeneous residual bootstrap p<0.05 (primary)
Reported local R2	out-of-sample gain (decisive); Jaccard≥0.75 (secondary)
Unbalanced designs	report $\pi_{\text{min}}$; $\Delta \theta_{\text{min}}$ understates the requirement, use the balanced value

## Limitations

The decline in accuracy as the subgroup coefficients become similar is not an unexpected failure but a predictable geometric consequence: as Δθ→0 the latent-score vectors of the two subgroups become nearly collinear and no criterion based on orientation can separate them. What this article adds is a quantification of where that boundary lies, given by the expression for $\Delta \theta_{\text{min}}$ and by the operating table that accompanies it.

PCA-PI-PLS-ABS is designed to detect directional heterogeneity and its identifiable boundary can be stated quantitatively. Because PLS-SEM standardises both latent scores, the slope of a segment is a correlation and its line lies within $\pm 45^{\circ }$ of the $\overset{ˆ}{\xi }$ axis, so the separation between two segments can never exceed $90^{\circ }$; a design whose requirement exceeds that ceiling is out of reach however large the sample. Within it, with $\Delta \theta_{\text{min}}$ defined as the smallest angular separation at which the mean accuracy reaches 0.80, the baseline condition of this study gives $26.69^{\circ }$, rising to $38.45^{\circ }$ at $\sigma_{\zeta }=0.30$, to $51.91^{\circ }$ at $\sigma_{\zeta }=0.50$ and to $76.60^{\circ }$ when weak loadings and large disturbances are combined. In terms of path coefficients, with equal groups and $\beta_{A}=1.0$, the largest separable $\beta_{B}$ ranges from 0.331 under favourable measurement to −0.615 under unfavourable measurement; below that boundary the segmentation accuracy falls to its chance level, which is the share of the largest group rather than one-half, and the method should not be used.

One caveat attaches to the expression itself and should be read before it is applied. Because the accuracy is scored on the plain scale, its chance level rises with group imbalance, so an unbalanced design crosses the 0.80 threshold at a smaller true separation than a balanced one. The exponent on $\pi_{\text{min}}$ is consequently positive, which means the expression understates the requirement when the minority segment is small: the 150:50 condition returns $21.34^{\circ }$ against $26.69^{\circ }$ for the same design when balanced, and the 180:20 condition had to be excluded from the calibration altogether because its chance level of 0.90 exceeds the threshold. $\Delta \theta_{\text{min}}$ should therefore be treated as calibrated for approximately balanced designs, and a researcher expecting a small minority segment should apply the balanced value instead of the value the expression returns.

Four further limitations follow from the validation. First, the procedure must not be applied when the aggregate relationship is itself null: partitioning an unstructured cloud raises the weighted local $R^{2}$ from 0.0057 to 0.3623, out of sample as well as in sample, which is why the homogeneity test is a required step rather than an optional check. Second, that test is conservative and does not reject when one subgroup has no relationship at all, since such a mixture is nearly observationally equivalent to a single population with larger dispersion; a non-significant result is therefore an absence of evidence rather than evidence of homogeneity. Third, with more than two latent classes the farthest-distance rule under-selects, retaining K=2 in 92% of replications when the truth is K=3 at β=(+1,0,−1), even though cutting the tree at K=3 recovers the classes with an accuracy of 0.8977; for K>2 the number of segments should be chosen by scanning K with the test and the stability index rather than by the dendrogram gap alone. Fourth, the empirical application shows that on real survey data the available angular separation may fall below $\Delta \theta_{\text{min}}$, in which case the method should be read as a detection tool for directional heterogeneity at the segment level rather than as a classifier of individual respondents.

## Ethics statements

This work did not involve human subjects, animal experiments, or data collected from social media platforms. The validation of the proposed method was conducted exclusively using Monte Carlo simulations with computer-generated data.

## CRediT authorship contribution statement

**Angga Dwi Mulyanto**: Conceptualization, Methodology, Software, Validation, Formal analysis, Investigation, Resources, Data Curation, Writing - Original Draft, Writing - Review & Editing, Visualization, Project administration, Funding acquisition. **Bambang Widjanarko Otok**: Conceptualization, Writing - Review & Editing, Supervision. **Jerry Dwi Trijoyo Purnomo**: Writing - Review & Editing, Supervision.

Supplementary material *and/or* additional information.

To ensure the reproducibility and transparency of the proposed PCA-PI-PLS-ABS method, the following materials are provided:

1. R Script (.R): The complete source code containing the algorithm functions and Monte Carlo simulation protocols.

2. Simulation Results Archive (.zip): A compressed folder containing raw output files (.txt) from Simulation 1 to Simulation 17, documenting the method's performance across various experimental conditions.

3. Online Computational Report: For ease of visualization and code inspection, a comprehensive web-based report (generated via R Markdown) is available at: https://rpubs.com/anggadwimulyanto/PCA-PI-PLS-ABS.

## Declaration of competing interest

The authors declare that they have no known competing financial interests or personal relationships that could have appeared to influence the work reported in this paper.

## Data Availability

I have shared the link to my data/code at the article.

## References

[1] Dash G., Paul J. (2021). CB-SEM vs PLS-SEM methods for research in social sciences and technology forecasting. Technol. Forecast. Soc. Change.

[2] Elston J.N., Pinto H.E.D.R.S.D.C., Nogueira C.F.S.V. (2026). Waves of innovation: the role of sustainability in driving impact in the blue economy – a PLS-SEM approach. Mar. Policy.

[3] Yamin M.A.Y., Al Aqra N.T.M. (2025). Achieving employee mental health through HR practices, spirituality and justice: empirical findings from Saudi Arabian health sector using PLS-SEM approach. Acta Psychol. (Amst)..

[4] Sarstedt M. (2008). A review of recent approaches for capturing heterogeneity in partial least squares path modelling. J. Model. Manag..

[5] Becker J.-M., Rai A., Ringle C.M., Völckner F. (2013). Discovering unobserved heterogeneity in structural equation models to avert validity threats. MIS Q..

[6] Astuti C.C., Otok B.W., Andari S. (2025). Segmentation of partial least squares structural equation modelling using kernel K-means clustering (PLS SEM KKC). MethodsX.

[7] N. Kock, “Using causality assessment indices in PLS-SEM,” 2022.

[8] Hair J.F., Hult G.T.M., Ringle C.M., Sarstedt M. (2022).

[9] Hamdollah R., Baghaei P. (2016). Partial least squares structural equation modeling with R. Pract. Assess. Res. Eval..

[10] Hadri Z.E.L., Abdem S.A.E. (2025). Finite iterative method based algorithm to estimate latent variables for reflective blocks in partial least squares structural equation modeling. Electron. J. Appl. Stat. Anal..

[11] Chin W., Cheah J.-H., Liu Y., Ting H., Lim X.-J., Cham T.H. (2020). Demystifying the role of causal-predictive modeling using partial least squares structural equation modeling in information systems research. Ind. Manag. Data Syst..

[12] Anekawati A., Otok B.W. (2017). Structural equation modelling with three schemes estimation of score factors on partial least square (case study: the quality of education level SMA/MA in Sumenep regency). J. Phys.: Conf. Ser..

[13] Otok B.W., Alfasanah Z., Aksioma D.F. (2026). Combination of partial least square structural equation modeling scheme of principal component analysis with importance performance analysis. MethodsX.

[14] Dukalang H.H., Otok B.W., Purhadi (2025). Modified partial least square structural equation model with multivariate adaptive regression spline: parameter estimation technique and applications. MethodsX.

[15] Lohmöller J.-B. (1989).

[16] Hair J.F., Hult G.T.M., Ringle C.M., Sarstedt M., Danks N.P., Ray S. (2021). http://www.

[17] Hasan M.M., Bala B., Yoshitaka A. (2015). 2015 4th International Conference on Informatics, Electronics and Vision.

[18] Cadima J., Jolliffe I. (2009). On relationships between uncentred and column-centred principal component analysis. Pak. J. Stat..

[19] Zhang Z., Huang S., Shi Z., Wang D. (2005). Linear projection based dimension reduction analysis in algebra space. Jisuanji Gongcheng/Comput. Eng..

[20] Pan H., Badawi D., Miao R., Koyuncu E., Cetin A.E. (2022). ICASSP, IEEE International Conference on Acoustics, Speech and Signal Processing - Proceedings.

[21] Choi S. (2005). Lecture Notes in Computer Science (including subseries Lecture Notes in Artificial Intelligence and Lecture Notes in Bioinformatics).

[22] Sauer T. (2019).

[23] Alipourfard N., Fennell P.G., Lerman K. (2018). WSDM 2018 - Proceedings of the 11th ACM International Conference on Web Search and Data Mining.

[24] Lerman K. (2018). Computational social scientist beware: simpson’s paradox in behavioral data. J. Comput. Soc. Sci..

[25] Kievit R.A., Frankenhuis W.E., Waldorp L.J., Borsboom D. (2013). Simpson’s paradox in psychological science: a practical guide. Front. Psychol..

[26] Mardia K.V., Jupp P.E. (2000).

[27] Fisher N.I. (1993).

[28] Miyamoto S. (2022).

[29] Gere A. (2023). Recomm-endations for validating hierarchical clustering in consumer sensory projects. Curr. Res. Food Sci..

[30] Trinchera L., Fichet B., Piccolo D., Verde R., Vichi M. (2011). Classification and Multivariate Analysis For Complex Data Structures.

[31] Hennig C. (2007). Cluster-wise assessment of cluster stability. Comput. Stat. Data Anal..

[32] A. Baratloo, M. Hosseini, A. Negida, and G. El Ashal, “Part 1: simple definition and calculation of accuracy, sensitivity and specificity,” 2015. [Online]. Available: www.jemerg.com. PMC4614595 26495380

